# A Density-Based Approach to Decompose Interaction Energies and Generate Descriptors in Periodic Systems

**DOI:** 10.1021/acs.jctc.5c01043

**Published:** 2025-08-26

**Authors:** Grzegorz Niedzielski, James G. M. Hooper

**Affiliations:** † Department of Theoretical Chemistry, Faculty of Chemistry, Jagiellonian University, Gronostajowa 2, 30-387 Kraków, Poland; ‡ Doctoral School of Exact and Natural Sciences, Jagiellonian University, Łojasiewicza 11, 30-348 Kraków, Poland

## Abstract

We discuss the use of a density-based energy decomposition analysis (EDA) scheme, which we call the pawEDA approach, for partitioning interaction energies computed in periodic chemical systems. The method requires the use of the projector augmented wave (PAW) scheme to treat the behavior of the variational electron orbitals near the atomic nuclei, and it functions by generating a “promolecule” whose (smoothed) valence pseudoorbitals reproduce the same valence electron density as the superposition of the underlying fragments. This construction mimics the behavior of some previously published EDA schemes, particularly that of the DEDA method, which was the first scheme to build a promolecule from density-based constraints. The pawEDA scheme effectively creates a two-step transformation from the fragments to the final system, where the electron density generally shifts smoothly through and/or near the fragment boundaries within each step, and its simplicity complements well other more well-established (and more elaborate) EDA schemes, especially when it is used to compare two or more chemically related systems. It also allows for the construction of two-state “Δ-functions” that accompany the steps, which can be built from the likes of the electron density (Δρ), the electrostatic potential (“ΔESP”), and the electron localization function (ΔELF).

## Graphical abstract

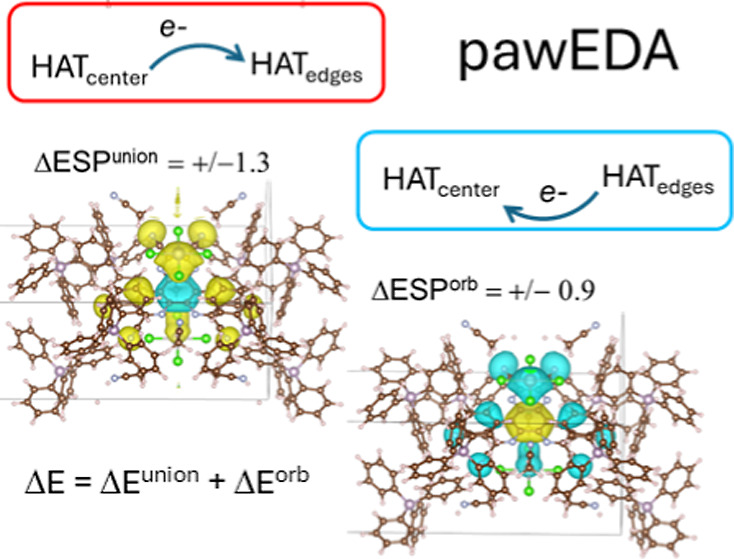

## Introduction

The concepts of “bonds” and “intermolecular interactions” are so central to our understanding of any system’s chemical properties that we often forget that the means we use to discuss them are not rigorously uniquely defined. Many of the terms and classifications that we use are more heuristic concepts than observable quantities. Still, the importance of in-depth understandings of both bonds and intermolecular interactions cannot be understated, especially as tools to compare similar systems or to predict the properties of new and/or otherwise poorly characterized systems; this is crucial in many fields of research like supramolecular chemistry,^1^ heterogeneous catalysis,^2^ or photocatalysis,^3^ and it is becoming even more important as the many varieties of machine-learning applications take root across all the fields of research that are involved in materials design.^4^ It is therefore apparent why there exists such a rich class of methods that try to analyze the electronic structure and quantize the energetic contributions that interacting fragments experience.^5–8^

Energy decomposition analysis (EDA) is an umbrella term for methods that try to decompose an energy of interaction into multiple contributions, usually into terms that somehow relate with the physical interactions that we expect to exist in the system. There are a plethora of EDA methods that span many fields of research and expertise, ranging from symmetry-adapted perturbation theory (SAPT)^5^ to orbital-based supermolecular approaches (ETS-NOCV,^6^ BLW-ED,^9^ ALMO-EDA,^7^ NEDA,^8^ and many, many others). Most of them are applicable to molecular systems, although some have been extended to periodic systems, and these have been gaining traction in recent years, where the earliest ones we are aware of are the PW-EDA,^10^ ALMO-EDA,^11^ pEDA,^12^ and Ensemble BLW-EDA^13^ methods. All of these methods are orbital-based or atom-centered approaches, but since density functional theory is the most commonly used method in such schemes it encourages the construction of density-based approaches, which is of course the core quantity of DFT. Such schemes have been developed too and have been implemented to study molecular systems, with the first one, to our knowledge, being the DEDA (i.e., “Density” EDA) method.^14^

Herein, we propose a simple method we refer to as pawEDA, which applies a density-based EDA approach to periodic systems. We employed it primarily because it is simple to execute within the popular Vienna Ab-initio Package (VASP),^15^ it decomposes the interaction energy into two logical contributions, and, in what was our original intent, it allows for visualization of certain descriptors, which we refer to here as Δ-functions, that give an insight into the nature of inter- and intramolecular interactions. The most popular of these Δ-functions is Δρ, which refers to a differential electron density between the system and either the fragments or some promolecule that is constructed from the fragments. We explore Δρ here along with two complementary differential functions: ΔESP and ΔELF, where ΔESP refers to a differential electrostatic potential (ESP) and ΔELF refers to a differential descriptor built from the Electron Localization Function (ELF).^16,17^ The features of the electrostatic potentials in different systems have long been discussed in the context of a chemical descriptor and they are still actively included in the key conclusions of many works in both molecular and surface chemistry;^18,19^ ΔESP/ΔMEP (MEP = Molecular Electrostatic Potential) functions have been explored recently too within our group, a generic version was first used by us in a periodic system to support observations from molecular models,^20^ and a representation of ΔMEP was built and analyzed as a standalone chemical descriptor for molecular interactions.^21^ The pawEDA method that we outline here can readily build such descriptors, and within the program we use, it is straightforward to extract information from the external (ionic) and exchange–correlation potentials. We describe how to use the pawEDA method, discuss its advantages and limitations, and showcase its use in several applications that involve molecular, periodic, and heterogeneous systems.

## Theoretical Approach

### The pawEDA Method

We start by describing the steps involved in generating all of the necessary stages of the pawEDA method, whose general framework is shown in Figure 1a. The steps are:

**Figure 1:**
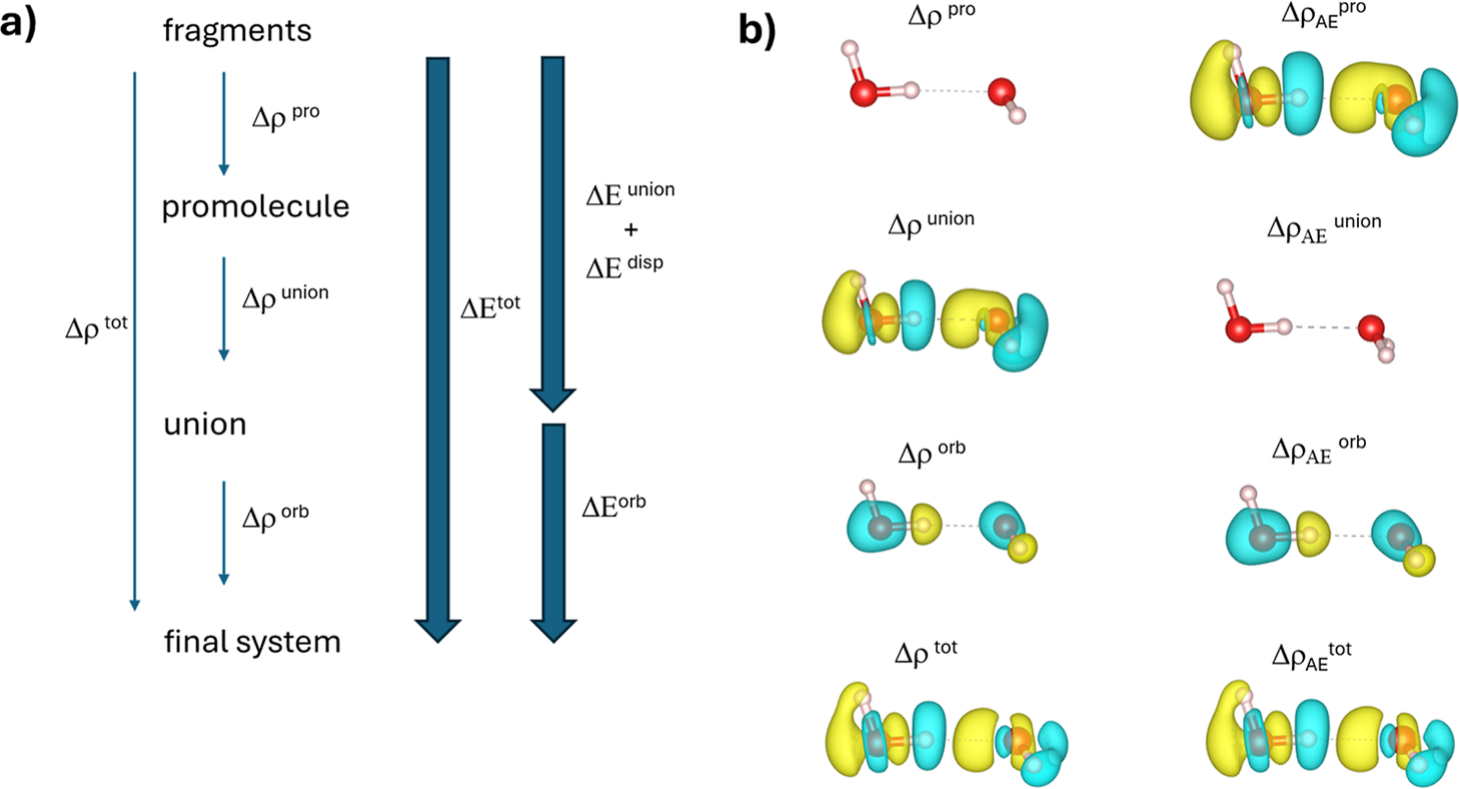
(a) A schematic of the three steps in pawEDA: fragments→promolecule, promolecule→union, and union→final system. The Δρ/Δ*E* functions are constructed by taking differences between the electron-densities/total-energies associated with the systems that comprise the steps, and the arrows show which systems are associated with the pro, union, orb, and tot labels that accompany those functions. (b) Isosurfaces (isovalue = 0.003 au) of the various Δρ functions for a water dimer. The yellow isosurface corresponds to a positive value and the cyan isosurface corresponds to a negative value.

#### Step 1—The Fragments

The pawEDA approach starts by separating the system into two or more fragments (each defined within the same periodic simulation cell that the system has) and then running single-point calculations on each individual fragment.

#### Step 2—The Promolecule

The pseudized (valence) electron densities from the fragments are then added together, wherein we employed a script that sums the constituent fragment electron densities at every grid-point (this created a new CHGCAR file for the VASP program, where CHGCAR is the file that stores the information about the electron density). It is important to stress at this point that the pseudized valence electron densities refer to the electron density that is generated from the auxiliary (smooth) orbitals that are variationally determined within the PAW method.^22,23^ This means that care must be taken to ensure that PAW one-center occupancies are supplied in order for VASP to accept the CHGCAR as input, and we tried supplying the PAW occupancies both from the individual fragments and from the final system, and in all cases reported herein, the final result was the same.

A “fixed-density” calculation is performed as invoked by the ICHARG = 11 tag in VASP, where the input (pseudized) valence electron density is kept fixed, and the program iteratively finds orbitals that reproduce it. This generates the reference pawEDA “promolecule” of the system, which is a model whose pseudized valence electron density is the same as the sum of the analogous electron densities from all of the individual fragments.

It is also important to note that within the PAW approach, the total electron density, ρ­(r), is not expressed solely as an “orbital density”, as is shown in eq 1

1 $$\rho \left(r\right)=\mathrm{\rho ̃}\left(r\right)+\sum_{a}\left[\rho_{c}^{a}\left(r\right)+\sum_{i,j}D_{ij}^{a}\left(\Delta \left(\phi_{i}^{a}\phi_{j}^{a}\right)\right)\right]$$

where ρ̃(*r*) is the orbital density that comes from the variationally determined orbitals (i.e., $\mathrm{\rho ̃}\left(r\right)=\sum_{n}f_{n}{\left|{\mathrm{\psi ̃}}_{n}\right|}^{2}$ , where *f*_*n*_ is the occupation number of orbital ${\mathrm{\psi ̃}}_{n}$ ), $\sum_{a}\left[\rho_{c}^{a}\left(r\right)\right]$ is the contribution that comes from the frozen core orbitals (which we do not consider here), and $\sum_{a}\left[\sum_{i,j}D_{ij}^{a}\left(\Delta \left(\phi_{i}^{a}\phi_{j}^{a}\right)\right)\right]$ contains the local (atomic) corrections that come about from expanding the smoothed and real wave functions with partial waves near the nuclei, see ref 23. Within PAW, the system orbitals, ψ_*n*_, are deconstructed such that they are substituted with smooth variationally determined auxiliary orbitals, ${\mathrm{\psi ̃}}_{n}$ , that are designed to match ψ_*n*_ only outside of spheres centered on each atom (where each sphere must be nonoverlapping and its radius is a key parameter of the PAW approach).^22^ The auxiliary orbitals are smoothed within each sphere in such a way that the features of the exact orbitals are recovered by projecting both the auxiliary and exact orbitals onto sets of atom-centered partial waves, ϕ_*i*_^*a*^ and ${\mathrm{\phi ̃}}_{i}^{a}$ , within each sphere. The total electron density must therefore take into account the difference between the true system orbitals and the auxiliary orbitals within the spheres, but the constraint we use here acts only on the orbital density, ρ̃(*r*).

The use of a density-based constraint during this stage nonetheless sets up the strong connection with the DEDA method.^14^ Within DEDA, the system responds to the constraint by locating orbitals that preserve the total density but minimize the contributions from the kinetic energy functional and the orbital-dependent nonlocal parts of the exchange–correlation functional. The pawEDA method’s promolecule functions similarly, except that the system has an additional way to respond to the constraints because of how the PAW method expands the wave functions with basis functions near the atomic nuclei, and this projection mechanism is what gives the system the ability to respond to the constraints on ρ̃(*r*). One limitation in this scheme is that the constraint that is used with the ICHARG = 11 keyword does not work with nonlocal contributions to the exchange–correlation functional, which prohibits the use of *meta*-GGA or hybrid functionals when using the default settings.

The top panel of Figure 1b shows differential electron densities between the promolecule that we obtained for a water dimer and the sum of the densities from the individual water molecules, where Δρ^pro^ (not shown) is the differential density constructed from the orbital density and Δρ_AE_^pro^ is the differential density that is associated with rebuilding the true (“all-electron”, AE) valence orbitals (locally computed on the same grid as Δρ^pro^). Here, it was seen that Δρ^pro^ is zero, as was enforced by the constraint that was used to build the promolecule, but Δρ_AE_^pro^ exhibits already a clear response that redistributes the electron density within each molecule. The origin of these changes in Δρ_AE_^pro^ is “local” in the sense that they come about mostly from compensatory changes in distributions of electron density near the atomic nuclei. For this reason, we show in Figure 2 how Δρ_AE_^pro^ changes along a cylinder that encapsulates the O–H···O contact, where the value of Δρ_AE_^pro^ at every grid-point within the cylinder is shown (as the cyan-colored data set) and the cylinder has a radius of ∼0.265 Å (0.5 bohr). The plot indicates two key responses: (i) there is an increase in density near the O atom and a decrease in density near the H atom of the O–H contact, and (ii) the density is disturbed near the hydrogen bond acceptor O atom (near 3.0 Å on the plot’s *x*-axis) such that there is a noticeable buildup of density directed along the hydrogen bond contact (this feature peaks around 2.0 Å on the plot’s *x*-axis). Thus, it is clear that this construction of the promolecule is already able to include redistributions of electron density that are mostly confined within the PAW spheres around each atom.

**Figure 2:**
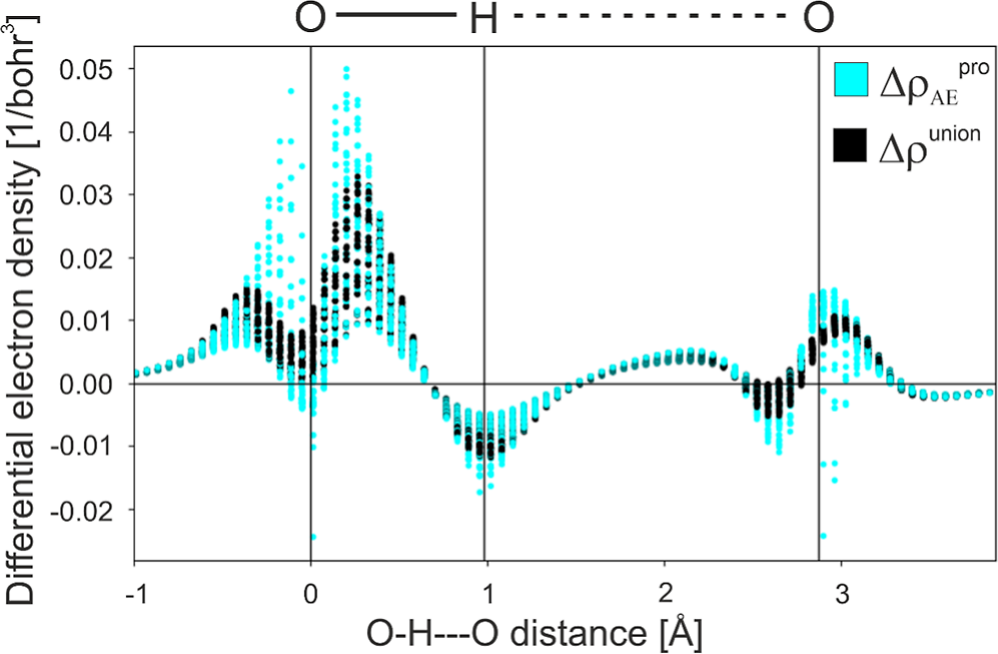
Absolute values of all the data points of Δρ_ΑΕ_^pro^ and Δρ^union^ within a cylinder of radius 0.5 bohr that encapsulates the O–H···O contact of the water dimer.

#### Step 3—The Union State

Next, the constraint of the electron density is relaxed, but the orbitals of the promolecule are reoptimized under the new constraint that the orbitals remain in the subspace that is spanned by the occupied orbitals from the promolecule. This generates what we refer to as the union state, and it is intended to be a model system whose orbitals best reproduce the superposition of the pseudo-orbital valence electron densities outside of the PAW spheres. It is technically enforced within VASP by using the IALGO = 4 input keyword and limiting the number of bands to the formal number of occupied orbitals in the system. The differential valence (pseudized) electron density that accompanies this step is shown in Figure 1b as Δρ^union^, and that which is obtained from the reconstructed (unsmoothed) system orbitals, Δρ_AE_^union^, was found to be the same. It is seen that the changes that were forced upon the system to accommodate the fixed-density constraints in the promolecule state seem smoothly transferred in this case to the auxiliary (smoothed) orbitals of the union state. Figure 2 shows explicitly how the locally computed contributions to Δρ^union^ and Δρ_ΑΕ_^pro^ differ from each other along the O–H···O contact, and here the same general shape is seen in both data sets, and the differences are largely confined to the attenuation of reconstructed densities near the nuclei.

#### Step 4—The “Orbital Interaction” State

Next, all of the constraints on the electron density and the orbitals are removed, and the system is allowed to relax normally. This is what we refer to as the “orbital interaction” stage because it allows the occupied orbitals of the union state to mix with the unoccupied ones, but it should be stressed that this does not directly correspond with the orbital interaction energies that are used in EDA schemes which explicitly use the fragment orbitals to gradually rebuild the interacting system. This is most easily seen in the Δρ^union^ differential density that was discussed above, where it is clearly seen that intrafragment polarization has already occurred. The features of Δρ^orb^ (and of Δρ_ΑΕ_^orb^ since it is the same) are shown in Figure 1b, and there it is seen that the features mostly “push back”, in a sense, against the responses that were forced upon the system in Δρ^union^.

An interesting perspective follows from considering how the “union” state was built, specifically in how the responses to the constraints localize predominantly within the PAW spheres. In a sense, this directs the “union” state to seek out a “tight-binding” representation of an intermediate state, by which we mean that the largest system responses comprise redistributions of density near the atomic nuclei. Thus, this “orbital interaction” step is also giving the system a chance to reverse these “tight-binding” responses, and that is what dominates Δ*E* ^orb^ in the case of the water dimer. This seems to effectively bias the system, in molecular systems at least, to create two “directions” of electron density upon progressing along the fragments → union → final stages. On the one hand, this loses some information that is expected of classical EDA schemes (due to their explicit use of fragment orbitals instead of densities, like separating out different types of polarization and charge-transfer processes), but, on the other hand, it can provide an interesting alternative in cases where the inductive vs charge-transfer vs Pauli repulsion contributions from orbital-derived schemes do not lead to clear rationales for chemical reactivity.

Figure 3 shows again all of the local (i.e., grid-point) values of the Δρ^union^ function within a cylinder that spans the O–H···O hydrogen bond within the H_2_O dimer (this data is also shown in Figure 2), as well as the complementary data from Δρ^orb^ and Δρ^tot^. The plot clearly shows how the contributions of Δρ^orb^ indeed push back against those of Δρ^union^. An interesting observation concerns what is observed near the middle of the hydrogen bond, near 2.0 Å on the *x*-axis of the Figure 3 graph. The buildup of electron density in this region is that which is also seen in the isosurface plots (Figure 1b), and its breadth is related in the literature with the “charge-transfer” character of the hydrogen bond. Of note here, then, is that this charge-transfer character is already present in the “union” state of pawEDA, which is similar in spirit to how the DEDA method was noted to predict a substantially diminished charge-transfer character vs other schemes when it was first implemented and tested with water dimers.^14^ This will be discussed further below.

**Figure 3:**
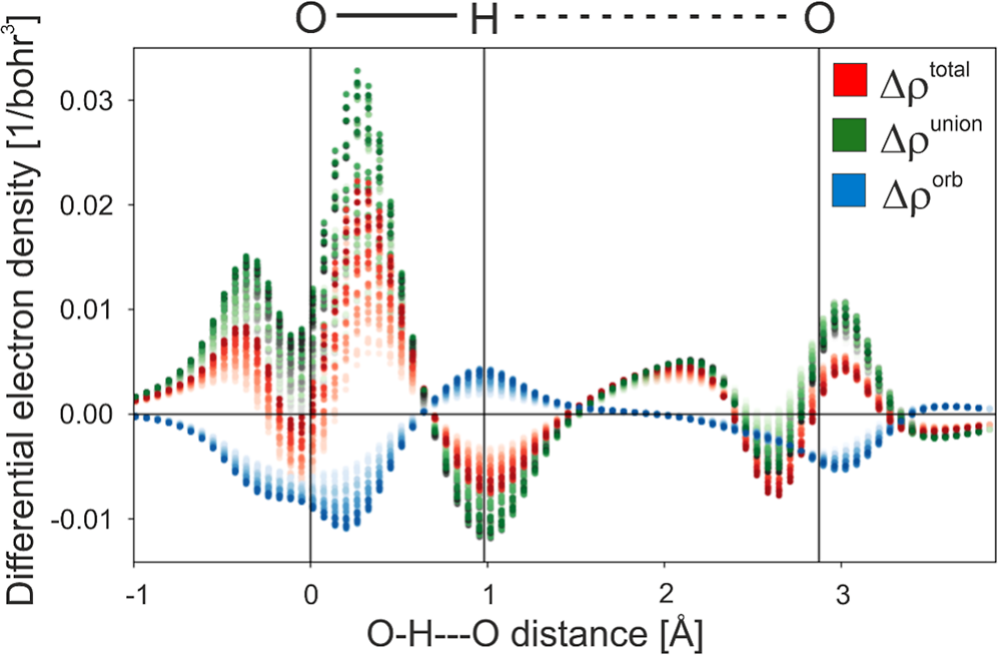
Absolute values of all the data points of Δρ^tot^, Δρ^union^, and Δρ^orb^ within a cylinder of radius 0.5 bohr that encapsulates the O–H···O contact of the water dimer. The data points are shaded such that the points with zero transparency are those which lie directly on the hydrogen bond axis, and the degree of transparency correlates with how far the data point is from the hydrogen bond axis.

### Computational Details

The calculations on periodic crystal structures were performed using density functional theory (DFT) methods within the Vienna Ab-Initio Simulation Package (VASP), version 6.4.2.^15^ Given the importance of noncovalent interactions between molecules and/or fragments, the PBE^24^ exchange–correlation functional was used with a D3-generation post-SCF dispersion correction^25^ (PBE+D3, including Becke–Johnson dampening). The plane-wave basis set cutoff was set to 500 eV, the C/N/O (2s/2p), P/Cl (3s/3p), Cu (4s/3d), Pd­(5s/4d), Pt­(6s/5d), and H (1s) valence electrons were treated explicitly and used in conjunction with PAW potentials^23^ that were supplied with the standard VASP package-version 6.4, and scalar relativistic effects were thus included within their implementation in the PAW potentials. The VESTA program was used to generate the Δ-function plots.^26^

## Applications and Analysis

### A Chain of Water Molecules

Figure 4a shows the model that we constructed of a periodic one-dimensional chain of water molecules, labeled **H**_**2**_ **O**_**chain**_, wherein the unit cell contains four molecules and was initially built from user intuition before the lattice constant was manually scanned in 0.01 Å increments and then set to the minimum value, of 8.830 Å, located along the scan (thus, this model is not expected to be a global minimum and the optimized coordinates are given in the ESI). The computed (molecule-averaged) interaction energy, Δ*E* ^tot^, of a water molecule within **H**_**2**_ **O**_**chain**_ is shown in Table 1, as well as the computed values of Δ*E* ^disp^, Δ*E* ^union^, and Δ*E* ^orb^ within the framework of the pawEDA method. Each water molecule is taken as its own individual fragment, and thus, the promolecule was built by taking the sum of the valence electron densities from each individual fragment. We also show the results for taking an optimized water dimer and placing it within a periodic box with 15 Å × 15 Å × 15 Å dimensions (this corresponds with the same system that we discussed earlier in Figures 1–3 and it is labeled **H**_**2**_ **O**_**dimer**_). The formulas for these energy contributions (using **H**_**2**_ **O**_**chain**_ as an example) are shown in eqs 2–5.

2 $${\Delta E}^{tot}=E\left(H_{2}O_{chain}\right)-\sum_{i}E\left(H_{2}O_{i}\right)$$

3 $${\Delta E}^{disp}=E^{disp}\left(union\right)-\sum_{i}E^{disp}\left(H_{2}O_{i}\right)$$

4 $${\Delta E}^{union}=E\left(union\right)-\sum_{i}E\left(H_{2}O_{i}\right)-{\Delta E}^{disp}$$

5 $${\Delta E}^{orb}=E\left(H_{2}O_{chain}\right)-E\left(union\right)$$

where *E*(X) is the total energy of system X, H_2_O_i_ is the *i*th water molecule in the model, *E* ^disp^(X) is the contribution of the post-SCF dispersion correction to the total energy of system X, and the “union” state is that which is described above (and shown schematically in Figure 1).

**Figure 4:**
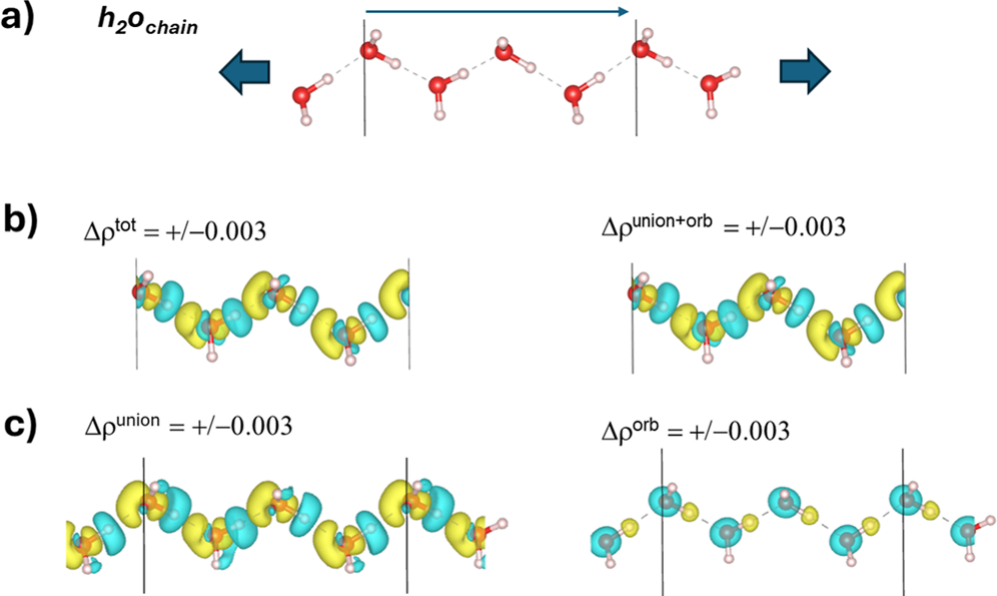
(a) The chemical structure of the **H**_**2**_ **O**_**chain**_ model. The unit cell contains four molecules, and the arrow and straight-line segments indicate the length of the lattice vector that defines the lattice constant associated with the periodic chain. (b,c) Isosurfaces (isovalues are shown on the figure in atomic units) of the various Δρ functions for **H**_**2**_ **O**_**chain**_. The yellow isosurface corresponds to a positive value and the cyan isosurface corresponds to a negative value.

**Table 1:** Total Interaction Energy (Δ*E* ^tot^) of the **H**_**2**_ **O**_**dimer**_ and **H**_**2**_ **O**_**chain**_ Models and Their Decomposition into Δ*E* ^disp^, Δ*E* ^union^, and Δ*E* ^orb^ within the pawEDA Approach (Left) and the DEDA Approach (Taken from ref 14)­^a^

pawEDA^a^				DEDA^a^	
	H_2_O_dimer_ /PBE + D3	H_2_O_chain_ /PBE + D3	H_2_O_dimer_ /PBE^c^		H_2_O_dimer_ /PBE^b^ ^,^ ^c^
Δ*E* ^tot^/eV (%)	–0.248 (100%)	–0.381 (100%)	–0.222 (100%)	Δ*E* ^tot^/eV (%)	–0.215 (100%)
Δ*E* ^union^/eV (%)	–0.215 (86.7%)	–0.317 (83.0%)	–0.211 (95.2%)	Δ*E* ^frz^/eV (%)	–0.152 (71.0%)
Δ*E* ^disp^/eV (%)	–0.018 (7.3%)	–0.025 (6.4%)	0.000 (0.0%)	Δ*E* ^pol^/eV (%)	–0.031 (14.5%)
Δ*E* ^orb^/eV (%)	–0.015 (6.0%)	–0.040 (10.6%)	–0.014 (4.8%)	Δ*E* ^CT^/eV (%)	–0.034 (15.9%)

a The energy difference is given in “eV per contact”, and the percentage values indicate the relative contribution each term makes to Δ*E* ^tot^.

b Taken from ref 14.

c Computed at the MP2/aug-cc-pVQZ-optimized geometry.

The binding energies in Table 1 correlate with the creation of one intermolecular hydrogen bond upon bringing the fragments together (expressed in eV per contact or equivalently in eV per molecule in the case of **H**_**2**_ **O**_**chain**_). Within pawEDA, the binding energies come almost entirely (>80%) from the contribution of Δ*E* ^union^. This confirms that the orbitals which recreate the superposition of (pseudized) fragment electron densities already well reproduce the self-consistently optimized orbitals of the systems, and it is consistent with the earlier comment that Δ*E* ^union^ can already implicitly include the contributions from electrostatic interactions between fragments, from Pauli repulsion, and, based on prior studies, from much of the charge-transfer and inductive character responses.

The reported DEDA decomposition^14^ of Δ*E* ^tot^ for the MP2/aug-cc-pVQZ-optimized geometry of the **H**_**2**_ **O**_**dimer**_ system is given on the right-hand side of Table 1, along with the analogous pawEDA decomposition of Δ*E* ^tot^ using the PBE functional without a dispersion correction (and using the MP2/aug-cc-pVQZ geometry in a periodic box with 15 Å sides). Here, it is seen that the Δ*E* ^union^ term from pawEDA comprises roughly 95% of the total interaction and is thus substantially larger in magnitude than the “frozen density” term (Δ*E* ^frz^) of the DEDA method, which comprises ∼71% of the total interaction energy. Within DEDA, the rest of the interaction energy, barring the BSSE correction, is decomposed using constrained density functional theory to separate out polarization (Δ*E* ^pol^) from charge-transfer (Δ*E* ^CT^); this shows that the Δ*E* ^union^ term of pawEDA indeed captures well over half of the contributions from the charge-transfer and polarization partitions within classical EDA schemes. In this, we note that this implementation of pawEDA does not strictly improve our physical understanding of this particular intermolecular interaction, although we note that a straightforward extension, which we intend to explore, is to use constrained DFT within pawEDA, as is done in DEDA, to build a “union” state without charge-transfer. A key feature of the simpler pawEDA scheme, however, is in how it decomposes the interaction into two contrasting shifts in electron density. We believe this can be most useful in cases where there are significant fragment orbital interactions that would shift electron density in different directions, for example, with forward and back-donations of electron density. This will be demonstrated later with a 3D molecular crystal and with H_2_ adsorbed to metal surfaces.

Our second aim of trying the pawEDA scheme was to facilitate the creation of various Δ-function descriptors, wherein the already discussed Δρ is certainly one of the most important and popular ones. The changes in electron density that accompany the states that are used to construct Δ*E* ^union^/Δ*E* ^orb^ are shown as Δρ^union^/Δρ^orb^ in Figure 4c, and Δρ^tot^ is shown in Figure 4b. The Δρ^tot^ and Δρ^union+orb^ plots both show the total differential (orbital) valence electron density between **H**_**2**_ **O**_**chain**_ and the sum of each of the four H_2_O fragments, as was enforced by the manner in which the promolecule was constructed. The Δρ^union^ differential density from Figure 4c, which accompanies Δ*E* ^union^, is seen to largely reproduce the features of the total differential density, as was expected from its dominant contribution to Δ*E* ^tot^. The Δρ^orb^ differential density shows a clear contrast to that of Δρ^union^, which is analogous to what was observed for **H**_**2**_ **O**_**dimer**_.

As was mentioned earlier, the differential (local) electrostatic potential (ΔESP/ΔMEP) has been shown to be a useful complementary descriptor, and Figure 5a shows how such functions look in the case of **H**_**2**_ **O**_**chain**_. Here, we refer to such plots with a generic ΔESP label, where the ΔESP plot contains the contributions to the local electrostatic potential from the external (nuclear) potentials, the electron–electron contributions that arise from the Hartree potential, and the local (or semilocal) contributions from the exchange–correlation functional. In the case of ΔESP_har_, the plot contains only the external (nuclear) potentials and the electron–electron contributions that arise from the Hartree potential. The ΔESP_har_^tot^ plot thus shows the differential function computed from the “classical” representation of electrostatic potentials (i.e., the electron–nuclear potential and the electron–electron contributions from the Hartree potential), and ΔESP^tot^ additionally includes the local contributions from the exchange–correlation potential, *V*_xc_.

**Figure 5:**
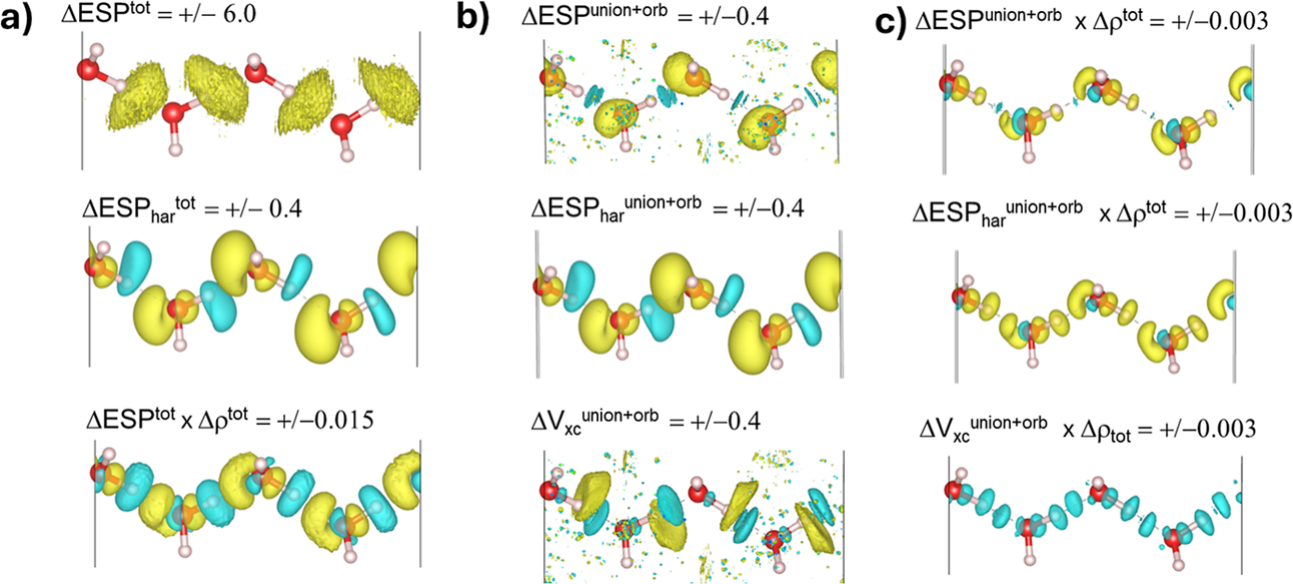
(a,b) Isosurfaces (isovalues are shown on the figure in atomic units) of the various ΔESP functions for **H**_**2**_ **O**_**chain**_. (a,c) additionally show a function where the local ΔESP is multiplied by the local value of Δρ. The yellow isosurface corresponds to a positive value and the cyan isosurface corresponds to a negative value.

The ΔESP_har_^tot^ plot in the middle panel of Figure 5a resembles the features that have been described for the ΔMEP function of molecular clusters,^21^ where the features loosely coincide with what is seen in Δρ^tot^. The ΔESP^tot^ plot (in the top panel of Figure 5a) becomes dominated by the contributions of *V*_xc_ near the fragment boundaries, and it becomes difficult to interpret beyond that. We note that the alleviation of such “strong” (perhaps better described as “appearing”) electrostatic interactions near the fragment boundaries is a much-discussed topic that relates best with the “Pauli repulsion” term that is included in most EDA schemes. With regards to visualizing ΔESP^tot^, a more appealing representation can be obtained by multiplying it by Δρ^tot^, as is shown in the bottom panel of Figure 5a. This both mediates the contribution of *V*_xc_ to regions of low density and restores important energetic information concerning the electron–nuclear interactions (whose symmetric contributions otherwise cancel off in ΔESP),^21^ and there we recover features recognizable as those in ΔESP_har_^tot^ but with some accentuations near the fragment boundaries.

Figure 5b shows the ΔESP^union+orb^ and ΔESP_har_^union+orb^ plots, which correspond to computing a differential electrostatic potential from the final system and the promolecule geometry. Here, it is seen that ΔESP_har_^union+orb^ is the same as ΔESP_har_^tot^, which helps to confirm the valid construction of the Δρ-conserving promolecule. Also interesting is the smoothing out of the total differential electrostatic potential (by which the use of “total” means that it includes contributions from exchange–correlation) in ΔESP^union+orb^, which now resembles what is seen for ΔESP_har_^union+orb^ except for some clear perturbations near the fragment boundaries. The difficulties in visualizing ΔESP^union+orb^ are seen to clearly relate with the difficulties in visualizing the local contributions from *V*_xc_ (see the bottom panel of Figure 5b), but this representation of the total electrostatic potential suggests a diminished influence at the interfragment boundaries vs that of the Hartree potential alone. This seems appealing within the framework of Kohn–Sham density functional theory, wherein it is hoped that the contribution of the exchange–correlation functional is small relative to those of the total energy functional. Figure 5c shows the result when the ΔESP functions are multiplied by Δρ^tot^, and here, it is seen that both pictures give the same general impression that the largest system responses are well localized on each individual water molecule. It is also interesting to see that the apparent influence of V_xc_ in these representations of ΔESP is to diminish the features near the fragment boundaries. The differential local contributions of the exchange–correlation potential (*V*_xc_) are easily extracted in this setup (as the difference between ΔESP^union+orb^ and ΔESP_har_^union+orb^), and the corresponding Δ*V*_xc_ functions are also shown in Figure 5b (and in [Fig fig5]c after being multiplied by Δρ^tot^). Overall, they are seen to “localize” well along the interfragment contacts. Altogether, the various Δ-descriptors shown in Figure 5 demonstrate that the pawEDA method can easily be used to generate such descriptors not only with respect to the construction of the system from the fragments but also within each of the pawEDA steps, which could be useful in various machine-learning schemes. We submit that such descriptors can also be useful in helping us to understand intermolecular interactions in various types of applications with periodic systems, as will be shown with the next two examples.

### A 3D Supramolecular Crystal

We previously used both molecular EDA schemes and periodic Δρ/ΔESP descriptors to study intermolecular interactions within a family of supramolecular crystals that had close contacts between the π-acidic HAT­(CN)_6_ molecule and adjacent [Pt­(X)_4_]^2−^ metal complexes.^20^ It was determined that the constituent [Pt­(Cl)_4_]^2–^ complexes in the **1-Cl** crystal structure engaged in stronger charge-transfer interactions than did the [Pt­(CN)_4_]^2–^ complexes in the analogous **1-CN** crystal structure. As a test of how such a system is described by pawEDA, the binding energy associated with introducing a single HAT­(CN)_6_ molecule to the unit cells of both crystal structures was reproduced here in both **1-Cl** and **1-CN** and decomposed with the pawEDA method. The results are shown in Table 2.

**Table 2:** Total Interaction Energy (Δ*E* ^tot^) for the **1-Cl** and **1-CN** Molecular Crystal Structures and Its Decomposition into Δ*E* ^disp^, Δ*E* ^union^, and Δ*E* ^orb^ within pawEDA

	1-Cl/PBE+D3	1-CN/PBE+D3
Δ*E* ^tot^/eV per cell	–5.30	–4.72
Δ*E* ^disp^/eV per cell	–3.53	–3.44
Δ*E* ^union^/eV per cell	0.22	0.29
Δ*E* ^orb^/eV per cell	–2.00	–1.57

The results show, first, that the overall binding energy of HAT­(CN)_6_ is dispersion-dominated, as was noted before, and, like what we saw with the ETS-NOCV^6^ EDA scheme, the main difference between **1-Cl** and **1-CN** concerns the orbital interaction energy, Δ*E* ^orb^, which is stronger in the case of **1-Cl**. We should stress that the Δ*E* ^orb^ terms of the two schemes (pawEDA vs ETS-NOCV EDA) are not directly comparable because they use differently constructed promolecules, but in this case, the results coincide because of the nature of the apparent charge-transfer. Figure 6a shows the total differential electron density, Δρ^tot^, for the **1-Cl** crystal structure and its Δρ^union^ and Δρ^orb^ decompositions. The plot of Δρ^tot^ matches what we reported before,^20^ with both periodic and molecular models, and Δρ^union^ and Δρ^orb^ nicely partition it into one feature, Δρ^union^, that appears to diminish electron density near the center of HAT­(CN)_6_ and another, Δρ^orb^, that appears to increase electron density near the center of HAT­(CN)_6_. The charge-transfer character that was seen within the ETS-NOCV method was seen in that scheme’s “orbital interaction” stage and was related specifically with an orbital interaction between the metal complex and HAT­(CN)_6_ that transferred electron density to HAT­(CN)_6_. The two schemes (the orbital interaction contribution in ETS-NOCV and the Δρ^orb^ contribution in pawEDA) thus qualitatively agree in the sense that there is an event of accumulated density near the center of HAT­(CN)_6_ and of depleted density from the metal complex. A caveat, though, is that the charge-transfer character of the contact is not so easily assigned in the pawEDA scheme, as the large inductive character of the Δρ^orb^ contribution is also evident from the spread of the Δρ and ΔESP functions over the whole HAT­(CN)_6_ molecule. The charge-transfer character does seem to be supported by the ΔESP^orb^ plot, which exhibits a clear contrast between the center of HAT­(CN)_6_ and all of its surroundings and this contrast remains visible in ΔESP^tot^, but, on the other hand, these features of ΔESP^orb^ are offset by the features of ΔESP^union^. Regardless, the contrast in features remains for the ΔESP^union+orb^ plot, so it is encouraging to see that the pawEDA method reinforces our prior finding that the electron density buildup over the center of the HAT­(CN)_6_ ring in **1-Cl** distinguishes it energetically from **1-CN**.

**Figure 6:**
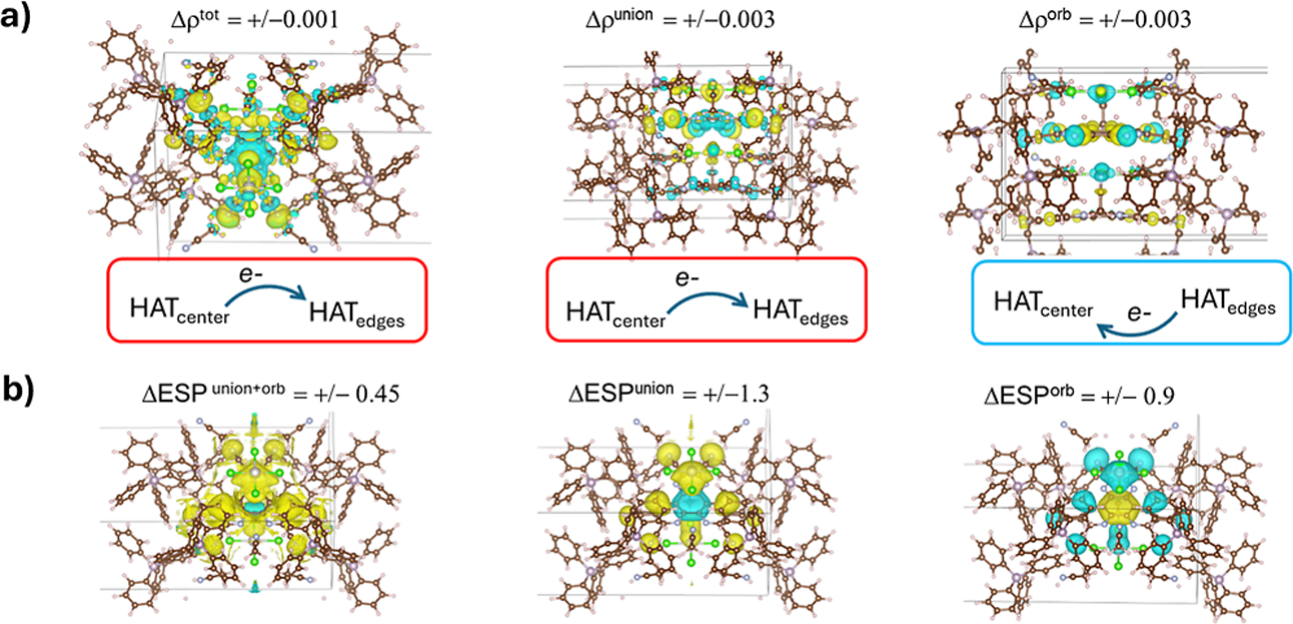
(a,b) Isosurfaces (isovalues are shown on the figure in atomic units) of the various local Δρ functions (part a) and local ΔESP functions (part b) for **1-Cl**. The yellow isosurface corresponds to a positive value and the cyan isosurface corresponds to a negative value.

We can also use the pawEDA promolecule to define a Δ-function from the electron localization function, the ΔELF function. The ΔELF function is used already as a descriptor of chemical interactions, for example, in studying how materials respond to external stimuli.^17^ With regard to supermolecular EDA methods, the ELF within regions of localized electron density can decay quite slowly with respect to distance, so adding the ELF functions of separate fragments together will usually result in summed functions whose values exceed 1.0, the theoretical limit of the ELF function. The pawEDA promolecule and the closely related “union” state consist of a single chemical system, within which one can compute the accompanying ELF function and use it to build a ΔELF function in the usual way (e.g., ΔELF^orb^ = ELF­(system) – ELF­(union)). We mention it here not particularly for its use in interpreting interactions between molecules but for its ability to diagnose the steps of the pawEDA procedure. The ΔELF function is zero within the ΔELF^union^ step of **1-CN** and **1-Cl** since the orbitals remain the same, so the ΔELF^union+orb^ function conveys information solely from the Δ*E* ^orb^ step, and this plot is shown in the Supporting Information (Figure S1). It shows mostly that the π-orbitals near the center of HAT­(CN)_6_ are perturbed, as well as the electron localization around the terminal CN groups.

### H_2_ Interacting with a Metal Surface

To explore the potential use of pawEDA in heterogeneous systems, we last use it on models of an H_2_ molecule interacting with either a Pd(001) or Cu(001) surface. We selected this system because it was one of the examples explored with an earlier periodic EDA scheme^12^ (pEDA) that makes use of a fragment orbital-based promolecule. The pEDA scheme confirmed earlier results of theoretical studies that concluded that Pauli repulsion is the determining factor in the stronger interaction of H_2_ with the Pd(001) substrate. Considering the altered construction of the promolecule in pawEDA, we were curious to see what the major differences between the two systems were with it. We used a pair of geometries that were reported in the pEDA study wherein the H_2_ molecule has a bond length of 0.78 Å and it is placed 1.60 Å above the top layer of the surface; in these models, only a two-layer asymmetric slab model is used, the Cu/Pd atoms are kept fixed at what would be their relative positions in their bulk phases, the H_2_ molecule is placed on one of the slab–vacuum interfaces, the length of the lattice vector that spans the vacuum layer between periodic slab images is 25 Å, and the *k*-points were sampled with a 4 × 6 × 1 Γ-centered Monkhorst–Pack grid (the results with higher 5 × 7 × 1 and 6 × 8 × 1 *k*-point meshes are shown in the Supporting Information).

The results from running the pawEDA scheme on the H_2_/Pd­(001) and H_2_/Cu­(001) systems are shown in Figure 7 and Table 3. Figure 7 shows the set of Δρ functions for both systems, and from these, it is clearly seen that their overall characters resemble one another. The Δρ^tot^ plot shows in general a diminished electron density profile around H_2_ and signs of polarization of electron density within the substrate. In these respects, some key differences between the systems are already visible: (i) the local redistributions of electron density within the substrate are more prominent in the case of Pd(001), particularly concerning regions of increased electron density, and (ii) the shape of Δρ^tot^ around H_2_ appears to skew more prominently away from the interface in the case of Cu(001). The decomposed differential densities, Δρ^union^ and Δρ^orb^, mostly segregate into one contribution, Δρ^union^, that diminishes the electron density around the atoms near the interface, and the second contribution, Δρ^orb^, restores electron density. Overall, the features around the Pd atoms are noticeably enhanced vs those around the Cu atoms, which manifest themselves quantitatively in the larger magnitudes of the computed Δ*E* ^union^ and Δ*E* ^orb^ values in the case of H_2_/Pd­(001). This leads to the somewhat intuitive picture that the Pd(001) model substrate is better able to polarize and localize electronic structural distortions within the region where Pd atoms make contact with the adsorbed H_2_ molecule, supporting the notion that H_2_ engages in more effective orbital interactions with the Pd(001) substrate.

**Figure 7:**
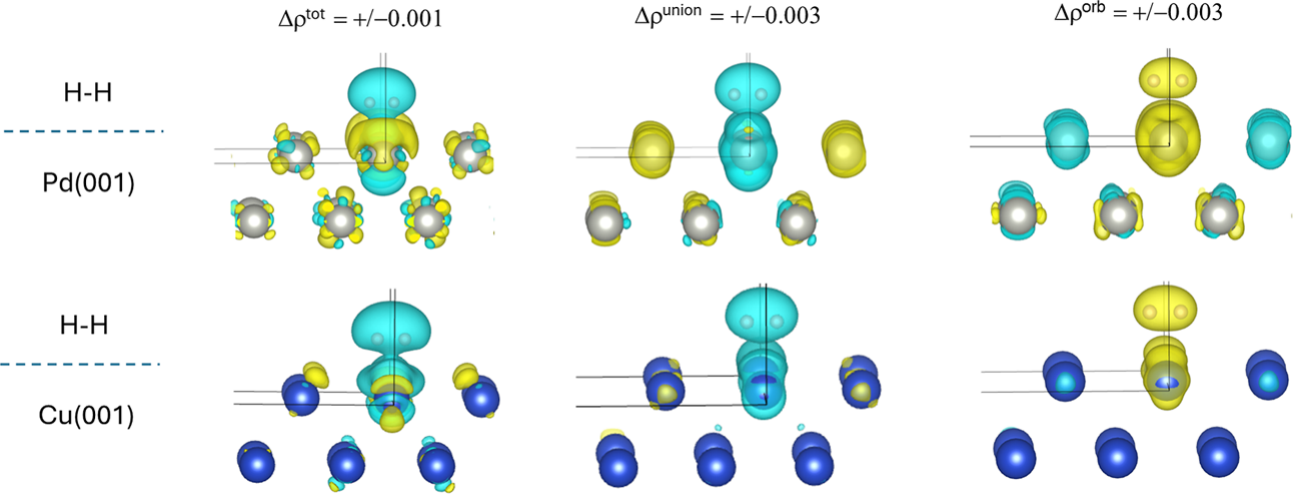
Isosurfaces (isovalues are shown on the figure in atomic units) of the various local Δρ functions for the **H**_**2**_ **/Cu­(001)** and **H**_**2**_ **/Pd­(001)** systems. The yellow isosurface corresponds to a positive value and the cyan isosurface corresponds to a negative value.

**Table 3:** Total Interaction Energy (Δ*E* ^tot^) for the **H**_**2**_ **/Cu­(001)** and **H**_**2**_ **/Pd­(001)** Systems and Its Decomposition into Δ*E* ^disp^, Δ*E* ^union^, and Δ*E* ^orb^ within pawEDA

	H_2_/Cu(001)/PBE+D3	H_2_/Pd(001)/PBE+D3
Δ*E* ^tot^ /eV	0.279	–0.353
Δ*E* ^disp^/eV	–0.157	–0.156
Δ*E* ^union^ /eV	1.285	1.742
Δ*E* ^orb^ /eV	–0.848	–1.947

When the union state is built, the orbitals are optimized under the constraint that they span the same subspace as those that were used to build the pawEDA promolecule. In the cases of **H**_**2**_ **O**_**chain**_, **1-Cl**, and **1-CN**, this leads to situations where the occupied orbitals of this state were the same as those of the promolecule. In the cases of **H**_**2**_ **/Cu­(001)** and **H**_**2**_ **/Pd­(001)**, however, we must also consider the influence of the band dispersion. In building the union state for an *N*-electron system in a spin-restricted formalism, the lowest *N*/2 orbitals were taken at each *k*-point that was used to generate the crystal orbitals of the promolecule; the union state thus loses some information in these metallic systems concerning the smeared occupancies of the energy levels near the Fermi level and the overlap of the bands as the underlying crystal momenta of the crystal orbitals are changed. This does not mean that the chosen orbitals in the union state were unaffected by band dispersion and smeared occupancies because their presence in the calculations of the fragments and promolecule rather ensures that they were in some way, but such effects must be considered when weighing the relative importance of either the Δ*E* ^union^ or Δ*E* ^orb^ pathway in the pawEDA scheme. To this end, the ΔELF^union^ and ΔELF^orb^ functions are shown for both systems in Figure 8, and here, it is clearly seen that the union states of the two systems respond very differently to the manner in which they were constructed. They do, in our view, reinforce the conclusions that were drawn above in the sense that in the case of Pd(001), the largest perturbations indeed occur around the H_2_ binding site, but in Cu(001), the surface states are much more perturbed. This corroborates that it is indeed the ability of Pd(001) to locally interact with H_2_ that best distinguishes it. The extent and accuracy in how this perturbation is depicted is another matter that must be explored, for example, the different “channels” of electron density may differ with alternate slab models, relaxed geometries, etc. However, we believe that interactions of chemical systems with metal surfaces may be the strength of this scheme precisely because it will seek out local inductive density responses over delocalized charge-transfer.

**Figure 8:**
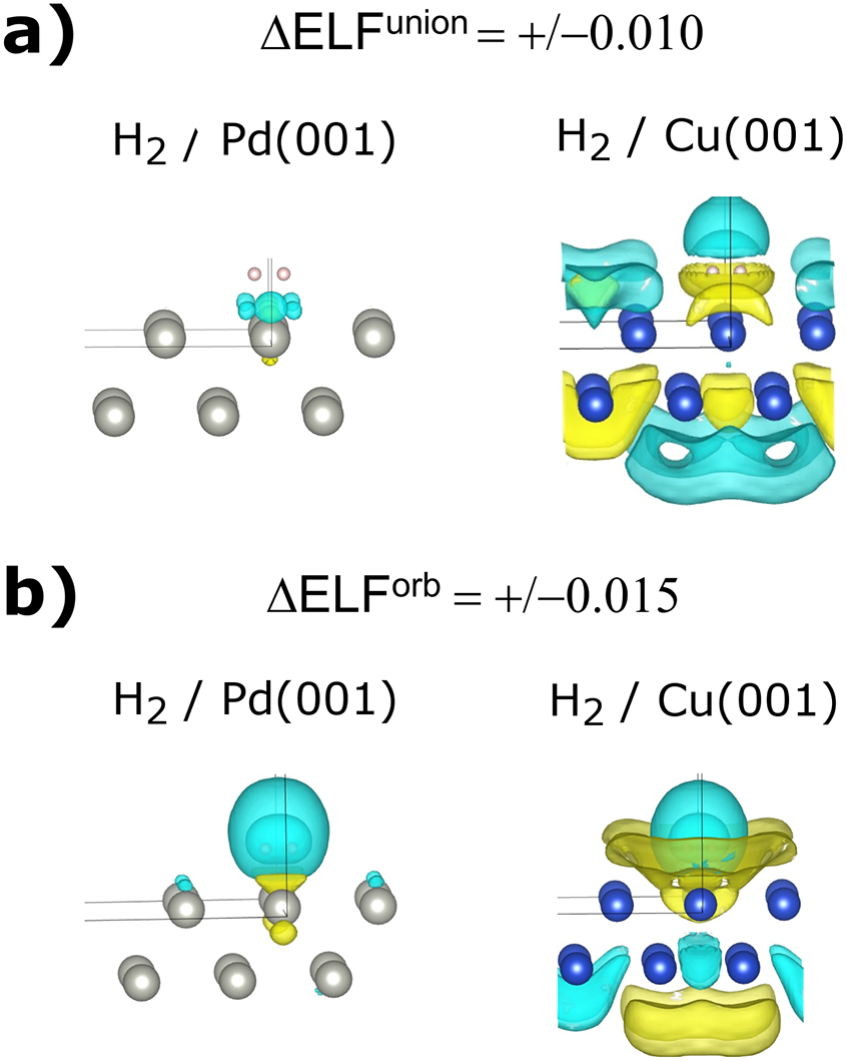
(a, b) Isosurfaces (isovalues are shown on the figure in atomic units) of the ΔELF^union^ function (part a) and ΔELF^orb^ function (part b) for the **H**_**2**_ **/Cu­(001)** and **H**_**2**_ **/Pd­(001)** systems. The yellow isosurface corresponds to a positive value and the cyan isosurface corresponds to a negative value.

## Conclusions

A method, which we coin pawEDA, has been presented and used to decompose interaction energies between user-specified fragments in periodic chemical environments. Although the scheme is simpler in setup than many commonly used energy decomposition analysis (EDA) schemes, we believe it complements them well by focusing on using the fragment densities, instead of the fragment orbitals, to build the transitory states between fragments and the final chemical system. It relies on using a PAW-construction of the system orbitals that permits the system to respond to constraints derived from the superimposed fragment electron densities (this response relates to the role of PAW projectors in rebuilding the “all-electron” valence orbitals near the atomic nuclei). The scheme seeks, in a sense, a “tight-binding” inspired intermediate whose current form already includes intra-/interfragment redistributions of electron density (i.e., induction/polarization/change-transfer). We suggest that constrained density functional theory could be used, as it is in DEDA, to separate polarization from charge-transfer.

In the cases we explored here, however, it was observed that the current scheme nicely separates out two “pathways” where the disturbances in electron density within each pathway appear to smoothly transition from one region to another, but, at the same time, the redistributions of electron density between the two pathways contrast sharply with each other. This was used to qualitatively visualize the important inductive character within intermolecular interactions of a one-dimensional chain of water molecules and within a supramolecular crystal that we previously studied. Another important aspect of the approach is that it allows us to build two-state Δ-function descriptors in periodic systems that are otherwise difficult to interpret when they are built from the fragments alone, namely, the ΔESP/ΔMEP and ΔELF functions that we address herein. This includes visualizing the influence of the exchange–correlation potential when building intermediate states, whose differential total electrostatic potentials resemble well those of the differential Hartree electrostatic potential alone. The method seems to be capable of functioning with heterogeneous systems, which we showed with the case of H_2_ binding to metallic Cu(001) and Pd(001) substrates, wherein we also suggest that the ΔELF is a useful diagnostic tool to help analyze the results.

In closing, the discussed pawEDA method is a simple but robust scheme that is capable of decomposing the total interaction energy of molecular, heterogeneous, and solid-state systems in periodic environments. Since it requires only the densities of the fragments, the density of the promolecule can be built also by manipulating the occupied orbitals of the fragments; for example, electronic smearing or altered fragment orbital occupations can be used in various scenarios, provided that care is taken to interpret the results in a physically meaningful sense. A key result of pawEDA is that it allows for the generation of some nonconventional descriptors (i.e., the Δ-descriptors referred to herein) that can be useful both for visualizing complicated bonding processes and for potential applications, for example, in machine-learning.

## Supplementary Material



## Notes

ΔELF and ΔV_xc_ plots for **1-Cl**, benchmarking information about the *k*-point sampling for **H**_**2**_ **/Cu­(001)** and **H**_**2**_ **/Pd­(001)**, and the structural coordinates of the H_2_O_dimer_, H_2_O_chain_, 1-CN, 1-Cl, H_2_/Cu­(001), and H_2_/Pd­(001) computational models (PDF)
