# Supplementary material for: A Density-Based Approach to Decompose Interaction Energies and Generate Descriptors in Periodic Systems
Source: J Chem Theory Comput. 2025 Aug 26;21(17):8446–55. doi: 10.1021/acs.jctc.5c01043 (PMC12424162; doi:10.1021/acs.jctc.5c01043)
Supplement: Supplementary file 1 [file ct5c01043_si_001.pdf]

Supplemental Information for:

A density based approach to decompose interaction energies and generate descriptors in periodic systems

*Grzegorz Niedzielski,[1,2] James G. M. Hooper[1]**

[1]Department of Theoretical Chemistry, Faculty of Chemistry, Jagiellonian University, Gronostajowa 2, 30-387 Kraków, Poland

[2]Doctoral School of Exact and Natural Sciences, Jagiellonian University, Łojasiewicza 11, 30-348 Kraków, Poland

Table of Contents:

ΔELF and ΔV_{xc} function of 1-Cl .... page 2

The dependence of the energy decomposition on the sampled k-points .... page 3

H_2O_{chain} in POSCAR format .... page 4

1-Cl in POSCAR format .... page 5

1-CN in POSCAR format .... page 19

H_2/Cu(001) in POSCAR format .... page 33

H_2/Pd(001) in POSCAR format .... page 34

ΔELF and ΔV_{xc} function of 1-Cl

The most interesting feature in the ΔELF^{orb} of **1-Cl** is the ‘disruption’ of the π-system in the central ring of $HAT(CN)_6$. The largest overall ‘disruption’ occurs rather near the terminal CN groups of $HAT(CN)_6$, which is intuitive because there is a substantial redistribution of electron density particularly near the edges of $HAT(CN)_6$ in this step. To complement this plot, a differential V_{xc} plot (multiplied locally by the differential electron density) is shown in Figure S1b to demonstrate that within pawEDA it also highlights some of the intermolecular contacts in this system, as was seen in the main text for $\mathbf{H_2O_{chain}}$.

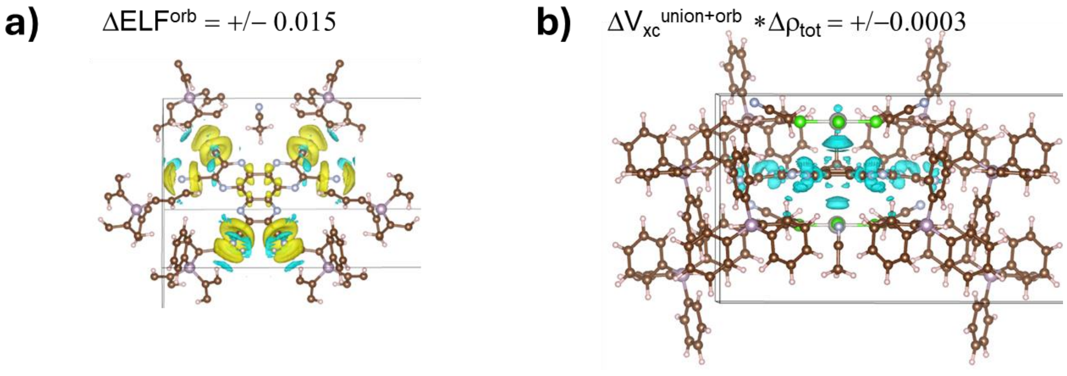

Figure S1. a,b) Isosurfaces (isovalues are shown on the figure in atomic units) of the ΔELF^{orb} function (part a) and local contributions to the $\Delta V_{xc}^{union+orb}$ function (part b) for **1-Cl**. The yellow isosurface corresponds to a positive value and the cyan isosurface corresponds to a negative value.

The dependence of the energy decomposition on the sampled k-points

The **H_2/Cu(001)** and **H_2/Pd(001)** results use restricted-spin fragments, where the fragments are the metal surface and the H_2 molecule. Table S1 shows how the binding energies change when more k-points are sampled (the main text presents the results with a 4x6x1 k-point mesh), and here is it seen that the results are fairly well converged with these particular surface models. We expect these results to be more sensitive when, for example, the slab is thicker and the metal atoms within it are allowed to relax, but we leave this to a future study.

	**H_2/Cu(001)**<br>**PBE+D3**<br>**4x6x1**	**H_2/Cu(001)**<br>**PBE+D3**<br>**5x7x1**	**H_2/Cu(001)**<br>**PBE+D3**<br>**6x8x1**	**H_2/Pd(001)**<br>**PBE+D3**<br>**4x6x1**	**H_2/Pd(001)**<br>**PBE+D3**<br>**6x8x1**
**ΔE^{tot} / eV**	**0.279**	**0.244**	**0.264**	**-0.353**	**-0.345**
**ΔE^{disp} / eV**	**-0.157**	**-0.157**	**-0.157**	**-0.156**	**-0.156**
**ΔE^{union} / eV**	**1.285**	**1.257**	**1.242**	**1.742**	**1.760**
**ΔE^{orb} / eV**	**-0.848**	**-0.856**	**-0.821**	**-1.947**	**-1.954**

Table S1. The total interaction energy (ΔE^{tot}) for the the **H_2/Cu(001)** and **H_2/Pd(001)** systems and its decomposition into ΔE^{disp}, ΔE^{union} , and ΔE^{orb} within the pawEDA approach. The header shows the model, the computational method (PBE+D3), and the dimensions of the grid that was used sample k-space.

H_2O_{chain} in POSCAR format

```
O   H   O   H   O   H   O   H
   1.000000000000000
    8.830120   0.000000   0.000000
    0.000000  20.000000   0.000000
    0.000000   0.000000  20.000000
O   H   O   H   O   H   O   H
1    2    1    2    1    2    1    2
Direct
  0.016714  0.321887  0.241299
  0.113152  0.305668  0.262770
  0.042763  0.361505  0.215859
  0.268448  0.277827  0.298376
  0.362899  0.298703  0.279641
  0.276111  0.281747  0.346599
  0.513283  0.333519  0.244593
  0.610612  0.329165  0.270389
  0.508704  0.379461  0.229249
  0.766831  0.317778  0.313737
  0.863431  0.320321  0.287062
  0.784307  0.343177  0.354301
```

1-Cl in POSCAR format

Note that the fragmentation we used comprised a single HAT molecule in the first fragment and the rest of the system in the second fragment. The first fragment corresponds to the first thirty atoms in the following structure.

```
N C N C Cl Pt N C H C H P
   1.000000000000000
   12.7350999999999992    0.0000000000000000    0.0000000000000000
    0.0000000000000000   29.6483999999999988    0.0000000000000000
    0.0000000000000000    0.0000000000000000   17.6753999999999998
  N    C    N    C    Cl   Pt   N    C    H    C    H    P
   12   18   36   54   16    4   12   24   36  192  160    8
Direct
 0.3856509632132910  0.2977414636891535  0.4109531829685680
 0.3686583795164635  0.2957771422031358  0.1388608665212267
 0.3866384536143497  0.3438988115690293  0.2760439472593856
 0.3879729637732854  0.3173924664712720  0.6023910789177251
 0.3882146343298050  0.3816374687825816  0.0108224652797425
 0.4143270280928820  0.4515842277030478  0.2054306159766120
 0.3856649265211130  0.2022634745694490  0.4109342772080993
 0.3684814310607568  0.2043018306458824  0.1387985912125085
 0.3866551002389184  0.1561281127880272  0.2759695878294642
 0.3880410168537478  0.1825979442560928  0.6023708342086761
 0.3882047839382038  0.1184348330842295  0.0107529091061463
 0.4143594695141528  0.0484679001000024  0.2053990023918285
 0.3788518280221370  0.2986380216973759  0.2750455022308416
 0.3696649945730318  0.2745574887627825  0.2058646210991556
 0.3810223115994361  0.2740599490240231  0.3462503607698196
 0.3892510533957864  0.3645976273998577  0.2093235544776542
 0.3854733582861055  0.2739781561607458  0.4747902711304584
 0.3805767836407987  0.3402554587517719  0.1401525423742147
 0.3847839551639082  0.3629795414990087  0.0688926130623353
 0.3870881316450405  0.2983207386511610  0.5445955692744876
 0.4026975690155022  0.4125316725183799  0.2083190707233077
 0.3788584142568971  0.2013889469808169  0.2750101804134690
 0.3696265843806172  0.2254792638763083  0.2058397505328158
 0.3810313577121822  0.2259503305897349  0.3462358090976429
 0.3892410416852942  0.1354569108250629  0.2092220316925612
 0.3854756531583723  0.2260221671061424  0.4747793215428810
 0.3804509010768872  0.1598269016944539  0.1400735589530249
 0.3846636436420814  0.1371178265464700  0.0687981332413242
```

0.3871083447680843	0.2016733055182496	0.5445781076159334
0.4027266902563585	0.0875224043480885	0.2082159353926780
0.1143354537486401	0.7022588038961252	0.9109307508550745
0.1315230524454722	0.7043017913436376	0.6387973174985110
0.1133454752699214	0.6561274336832578	0.7759679766804161
0.1120140250562806	0.6826000633328216	0.1023794673084701
0.1117994666611826	0.6184381799719041	0.5107494112691368
0.0856446444462317	0.5484675060026892	0.7053956219724741
0.1143486779678030	0.7977366103977396	0.9109572042222350
0.1313371764396256	0.7957782232918746	0.6388616354524754
0.1133616933042659	0.8438981393718322	0.7760456969787309
0.1119723876861944	0.8173947552862399	0.1023826136410066
0.1117816182169682	0.8816409773823267	0.5108257130346648
0.0856692965929621	0.9515839743162006	0.7054337888471789
0.6143490098407427	0.7977367245901635	0.5890428910603661
0.6313368885342437	0.7957782464636205	0.8611384610607014
0.6133620451422497	0.8438981986194674	0.7239546832157527
0.6119726654991477	0.8173963332059540	0.3976189152965323
0.6117823712851267	0.8816397098581931	0.9891760085445660
0.5856716081428497	0.9515842877581494	0.7945558033401577
0.6143352025825874	0.7022589150403182	0.5890691667450408
0.6315233337349977	0.7043018251876506	0.8612025873712091
0.6133451063703346	0.6561275015427549	0.7240316799522365
0.6120138968673913	0.6826016327894929	0.3976191481452663
0.6117985814405984	0.6184370165706009	0.9892490503244075
0.5856419712933387	0.5484679485691438	0.7946144835054397
0.8856514742703943	0.2022589371237413	0.0890457605525608
0.8686607566519250	0.2042228682168651	0.3611392926107851
0.8866368296374781	0.1561009260078202	0.2239559312012423
0.8879713965546199	0.1826020723499152	0.8976143800913653
0.8882171993708894	0.1183572887416243	0.4891730929237780
0.9143235727121101	0.0484155053711969	0.2945644523753941
0.8856645890850722	0.2977368212633297	0.0890662925105501
0.8684788958369423	0.2956979750982177	0.3612011652427400
0.8866568113492613	0.3438716749713470	0.2240305215497918
0.8880428794274822	0.3173966274351859	0.8976236873038305
0.8882018343641064	0.3815597209687337	0.4892505739816191
0.9143628920381489	0.4515315176088149	0.2946061570219933
0.1211407980004191	0.7013884380265338	0.7750078391575903
0.1303727063635632	0.7254788197614940	0.7058389586527158
0.1189673359065535	0.7259481414089852	0.8462348100100118
0.1107589689524607	0.6354565929789837	0.7092212748340796
0.1145223040993107	0.7260134842893285	0.9747788325596251
0.1195513817877620	0.6598270827180963	0.6400723563567573

0.1153407338807145	0.6371187734983099	0.5687965969383835
0.1129052936152288	0.7016658052201303	0.0445784089947826
0.0972752111875905	0.5875220161756445	0.7082142149227064
0.1211491253269993	0.7986375722291749	0.7750473822595678
0.1303352391163927	0.7745572855003416	0.7058640613387178
0.1189793786848449	0.7740578997621640	0.8462517418002952
0.1107488043970484	0.8645977369127386	0.7093247359452818
0.1145274642404284	0.7739693843343737	0.9747915243796912
0.1194193864256719	0.8402564900217817	0.6401540049921316
0.1152116386093492	0.8629810030771793	0.5688941702439768
0.1128979176213982	0.7983137966630574	0.0445950722284078
0.0973007953655703	0.9125314883027321	0.7083210500540085
0.6211491521349107	0.7986376344694400	0.7249527346084607
0.6303351472366069	0.7745573690460359	0.7941360332172112
0.6189795031250622	0.7740580121924083	0.6537483573533172
0.6107487821756551	0.8645977557135149	0.7906758007266099
0.6145277103873531	0.7739694535729500	0.5252085677780887
0.6194189253794269	0.8402565279146487	0.8598464865668222
0.6152110419408909	0.8629805436633802	0.9311067464011501
0.6128982080608150	0.7983142409086647	0.4554053883237363
0.5973016664948047	0.9125315387374057	0.7916768436473518
0.6211407560795970	0.7013884875178027	0.7249920555367226
0.6303728487564262	0.7254789046428650	0.7941609602855852
0.6189671839715987	0.7259482504609799	0.6537650933597442
0.6107590222947044	0.6354566225929758	0.7907782262955478
0.6145220438838308	0.7260135523154586	0.5252211121733389
0.6195518017657263	0.6598271496374786	0.8599272400234667
0.6153407086807552	0.6371184958122079	0.9312027848283873
0.6129049823022845	0.7016662380484255	0.4554211195256352
0.5972743086470300	0.5875220115253151	0.7917878754173356
0.8788518108934248	0.2013617550663231	0.2249537131261603
0.8696653678076411	0.2254426403337968	0.2941355838427152
0.8810223704635474	0.2259399638586724	0.1537490647773820
0.8892505095900261	0.1354025760935520	0.2906770837186607
0.8854739723431835	0.2260228778622811	0.0252092631167413
0.8805796234609188	0.1597445462929745	0.3598476624718264
0.8847884043291021	0.1370195671671423	0.4311067840583157
0.8870874891012669	0.2016788145849967	0.9554052391610667
0.9026962787577881	0.0874681932172606	0.2916800475350022
0.8788584866126510	0.2986108385838398	0.2249904619718679
0.8696263367326474	0.2745208252291684	0.2941599933677990
0.8810313065389321	0.2740496973517041	0.1537644361075123
0.8892414186205579	0.3645430974605271	0.2907773347641224
0.8854758941948562	0.2739788185263538	0.0252207269413422

0.8804475684367233	0.3401727593222974	0.3599260065313090
0.8846573431389425	0.3628802389867973	0.4312023701351721
0.8871090090367816	0.2983262832424441	0.9554206857669534
0.9027283392671431	0.4124772330731274	0.2917853216114202
0.6310349670624585	0.3281514934114944	0.1450067467520107
0.6311027593240138	0.1718480544279659	0.1450087886525731
0.6240800378731004	0.2499903505970803	0.2753326216780627
0.6313246215781276	0.2500049833537097	0.0139780783024665
0.8689045486452327	0.6718702542932351	0.6450233261703695
0.8689609484819668	0.8281707941240585	0.6450000699909381
0.8759230414213768	0.7500356235899296	0.7753396660348716
0.8686776699185574	0.7500181769243528	0.5139832310574918
0.3689610677196413	0.8281690350470575	0.8549991545562881
0.3689038697912677	0.6718684759175920	0.8549768765695857
0.3759227503346241	0.7500343644338446	0.7246600429028781
0.3686773507206763	0.7500167142669922	0.9860163945333384
0.1310306800611101	0.1718418624990092	0.3549960833188456
0.1311052252899867	0.3281453031724115	0.3549889237596542
0.1240798096660119	0.2499987506185881	0.2246676416174761
0.1313238621745708	0.2499885471710083	0.4860221040898671
0.6297307279025804	0.2500000432490018	0.1443852979805579
0.8702572675971953	0.7500110638973960	0.6443874177272347
0.3702568339820593	0.7500102317994832	0.8556113046355097
0.1297158533752683	0.2499962019200739	0.3556149125598923
0.5363802186317062	0.4255860582569923	0.8620254322454414
0.9635828387413743	0.5744029181065543	0.3620228354020336
0.4636114604364812	0.9255796897694822	0.1379902148226478
0.0363871374207843	0.0744119038542195	0.6379690826082526
0.4635795321133055	0.5744002707543711	0.1379705956367652
0.0364214871418970	0.4255912235647635	0.6379918158695091
0.5364288620128420	0.0744066938636935	0.8620133673277210
0.9636082204726780	0.9255822718935526	0.3620164627476356
0.6346381660569780	0.2501433093701939	0.5381032394153862
0.8653621394062786	0.7498613793123170	0.0381028022433517
0.3653621935121700	0.7498625853347937	0.4618964164907772
0.1346385427183535	0.2498467257068311	0.9618979846634805
0.5708872935546212	0.3938621319827551	0.8922064146614520
0.6146504350755062	0.3545752402534498	0.9290835533186100
0.9290967976456674	0.6061311949804991	0.3922027204859309
0.8853482278133200	0.6454225639551450	0.4290761646581885
0.4291104001228219	0.8938583568712338	0.1077977614573951
0.3853514699075791	0.8545738361883395	0.0709114481440761
0.0708895023821060	0.1061379226246262	0.6077911916496087
0.1146486464543441	0.1454263712176137	0.5709168287780144

0.4290953873211153	0.6061302722721679	0.1077946018414463
0.3853484402119064	0.6454230063708605	0.0709242449923745
0.0709051145153057	0.3938661085425636	0.6078008558935037
0.1146515189265399	0.3545772667972435	0.5709191025607359
0.5709074297854927	0.1061339682678072	0.8922013733725591
0.6146499189848862	0.1454243411012368	0.9290806492136402
0.9291089493050904	0.8938592519694419	0.3922050570862515
0.8853517194890379	0.8545733639004842	0.4290881081220244
0.8399313681059113	0.2486125649889692	0.5415099233249582
0.7262499584930149	0.2495219609365636	0.5401464105649750
0.6600687316658981	0.7513870385533146	0.0415096249836441
0.7737502564711077	0.7504797927613278	0.0401460425687350
0.1600687104071343	0.7513867311064786	0.4584901367977501
0.2737502889981452	0.7504801512748532	0.4598535239493985
0.3399312383119451	0.2513913335629365	0.9584903672770257
0.2262500171794679	0.2504756716828929	0.9598541174562698
0.6934527145937237	0.3475217457155682	0.9066982163163035
0.5647916137465065	0.3248824146766678	0.9196825691748950
0.6201494875193720	0.3593508147218115	0.9906705099929939
0.8065370331190551	0.6524738380500328	0.4067047507306751
0.9351979620942356	0.6751142902090682	0.4196414260637442
0.8798768939792977	0.6406560634177803	0.4906663782895737
0.3065452889505524	0.8475193030790661	0.0932889350842160
0.4352086588087360	0.8248801201397680	0.0803096322538587
0.3798587833805043	0.8593511881771612	0.0093245255989389
0.1934510289135573	0.1524808195322631	0.5933014365420828
0.0647884476282665	0.1751185359495323	0.5803192916352451
0.1201466553770673	0.1406531300657978	0.5093289449013779
0.3065377525472293	0.6524746079245035	0.0932963362849008
0.4351996015251338	0.6751141065394570	0.0803604932385331
0.3798766797954407	0.6406580899819930	0.0093336543838922
0.1934662482947765	0.3475255247206546	0.5932838241802643
0.0648036442139697	0.3248845641952719	0.5803508582343475
0.1201182951667651	0.3593461833146854	0.5093291221114309
0.6934646126118232	0.1524770511375476	0.9067164798752054
0.5648004880314881	0.1751163858794156	0.9196472252932656
0.6201154611939999	0.1406577176154982	0.9906713663025696
0.8065459849891413	0.8475185283837404	0.4067099902585933
0.9352102765091495	0.8248802936467856	0.4196884653381689
0.8798585207497134	0.8593491114163044	0.4906755110202229
0.8703233604500299	0.2458847318989093	0.4833385736807894
0.8701128452361596	0.2798098249601097	0.5671199331541316
0.8671409129627200	0.2195254597514822	0.5747361486092502
0.6296762882912196	0.7541140953958410	0.9833383086891464

0.6298895026775150	0.7201898131434518	0.0671210838388419
0.6328572857498879	0.7804742710172902	0.0747347657007248
0.1328564280456404	0.7804738141225992	0.4252649734591838
0.1296763407163556	0.7541136646882124	0.5166614940337262
0.1298902201206915	0.7201893448489575	0.4328788073304419
0.3703223154123751	0.2541213468703579	0.0166616902612128
0.3701205957885294	0.2201951705816828	0.9328811813103395
0.3671331461113149	0.2804793923722354	0.9252633573201942
0.8120175527401143	0.4787277862249258	0.5662348641780965
0.6198426415786090	0.3412897939655131	0.4138577997283339
0.7065492664603906	0.4814376952304419	0.5896622834407904
0.6230879415736094	0.3691385455654126	0.3501854535652208
0.6763710682425693	0.3891143466226076	0.6535237605422054
0.7832708150145441	0.3855436448279944	0.6715581991131737
0.4949225457693017	0.4437370734075646	0.6163615125557658
0.8760691458553144	0.5167089986192306	0.5715097653820883
0.6217674283815431	0.3598536385806066	0.4862071501483555
0.4110518153801617	0.4361959920856763	0.5665425166299284
0.2901347284521302	0.4629552226160047	0.6623380092555278
0.6653253716639930	0.5226138056252988	0.6154210766422505
0.6273506995475452	0.4068413349870670	0.4949313766210437
0.4762291271996702	0.4602269296454831	0.6897355755781510
0.6306692615772015	0.4350098301485274	0.4311310677455150
0.3738551719771873	0.4699983134977197	0.7121047734237759
0.3089035920149305	0.4458811133887010	0.5899415882186076
0.6043445400456503	0.3572629824979361	0.6812571693178987
0.6284458730958296	0.4159281584880792	0.3589781416731112
0.7295964871691548	0.5607155789174997	0.6185677673554627
0.8350903571124269	0.5575601474605741	0.5975800021528633
0.6397181609964075	0.3217353959697574	0.7260955926391656
0.8172231413591650	0.3502310841293489	0.7174958261868875
0.7459961221152149	0.3182324778061983	0.7444316595216319
0.6879835094091213	0.5212750084721497	0.0662322666200368
0.8801563474820774	0.6587113265449717	0.9138568533340372
0.7934504690445702	0.5185648895385291	0.0896599916906737
0.8769131414024205	0.6308627135048845	0.8501843883268937
0.8236282060624841	0.6108868118537570	0.1535238774710743
0.7167276992249428	0.6144575734777847	0.1715573451088914
0.0050769763955096	0.5562624816329089	0.1163609425499351
0.6239333232995825	0.4832936234167181	0.0715049980437418
0.8782318148139785	0.6401476502806386	0.9862064536620823
0.0889475008598297	0.5638032035531921	0.0665416353723107
0.2098647334364158	0.5370461077429549	0.1623389589034273
0.8346724504714359	0.4773888806096073	0.1154188523685288

0.8726497497349233	0.5931599838059524	0.9949312222266707
0.0237702693370497	0.5397733274482430	0.1897356192009528
0.8693310717297492	0.5649917529203428	0.9311307157701099
0.1261443723847006	0.5300026599426666	0.2121054709887976
0.1910958240991699	0.5541188119857917	0.0899414905392103
0.8956564765689305	0.6427368291455866	0.1812592772429625
0.8715551478196155	0.5840730943902159	0.8589775606089339
0.7704030979187598	0.4392868398873916	0.1185664075215540
0.6649100901426864	0.4424428498188917	0.0975769402188161
0.8602826750983007	0.6782644838022561	0.2260973843410754
0.6827746415870100	0.6497705406683695	0.2174935840753086
0.7540039921497447	0.6817683752890742	0.2444298197606536
0.1879804661451239	0.9787297640998214	0.4337640564302787
0.3801772636304565	0.8412930127118106	0.5861396154373867
0.2934476269255966	0.9814433026870010	0.4103368963069230
0.3769020088149691	0.8691386790050268	0.6498165222946575
0.3236165288520216	0.8891148403702386	0.3464895249380187
0.2167195222842630	0.8855488164968615	0.3284427147821471
0.5050757558594974	0.9437389737419009	0.3836374651934109
0.1239287136075040	0.0167103768703638	0.4284902096512914
0.3782741718072485	0.8598638216193653	0.5137950609186107
0.5889469247560378	0.9361956224013096	0.4334563008893958
0.7098651665420374	0.9629541547876007	0.3376605596150643
0.3346744423427238	0.0226181108025784	0.3845759343437152
0.3726506712175180	0.9068510009139731	0.5050753900705363
0.5237702186607418	0.9602279715592674	0.3102626182706004
0.3693155677610276	0.9350145103953401	0.5688796035903626
0.6261452850269088	0.9699977358367090	0.2878935221907907
0.6910956220586427	0.9458797562712050	0.4100568880470395
0.3956402669798405	0.8572623892342722	0.3187525862342439
0.3715381646552999	0.9159281716218998	0.6410298893860876
0.2704014865604805	0.0607186132260298	0.3814305915629216
0.1649083622300464	0.0575611448105485	0.4024204047670715
0.3602666828884955	0.8217320758609782	0.2739174765667087
0.1827664625229164	0.8502400005845083	0.2824925362702553
0.2539902414033160	0.8182334437820993	0.2555682630742472
0.3120175127939036	0.0212770485525057	0.9337684429870472
0.1198408904110744	0.1587120080031586	0.0861433710422479
0.2065495828779464	0.0185663190606990	0.9103416389838698
0.1230854101668272	0.1308631962311720	0.1498152899320887
0.1763705273459558	0.1108869273135707	0.8464742701561898
0.2832705855995319	0.1144571062052275	0.8284395456117176
0.9949227769269887	0.0562636821606214	0.8836393519372450
0.3760680730478114	0.9832959819649251	0.9284952340220081

0.1217663405563620	0.1401485125171295	0.0137935401297303
0.9110519323553565	0.0638043943506356	0.9334583136136304
0.7901346615158872	0.0370457032689173	0.8376621898613148
0.1653275523956715	0.9773894903494793	0.8845863697808114
0.1273489140555051	0.0931608548938803	0.0050691495622741
0.9762292809330776	0.0397745963072368	0.8102649679007684
0.1306676870782210	0.0649921098451946	0.0688691154414234
0.8738554855582661	0.0300025715838561	0.7878957265320297
0.8089035775463487	0.0541196780766991	0.9100588017212714
0.1043437009463896	0.1427384837573965	0.8187415534655827
0.1284435690745661	0.0840735392748794	0.1410220495466425
0.2295979339377004	0.9392878500882174	0.8814392624165969
0.3350915071338527	0.9424443324023333	0.9024257623086918
0.1397182669682181	0.1782660073683308	0.7739035046452191
0.3172228427837570	0.1497690238426460	0.7825007105190903
0.2459960631012837	0.1817683004851298	0.7555665019459115
0.1879805408801975	0.5212748638877432	0.4337660346549589
0.3801532960526342	0.6587112569783750	0.5861434043763448
0.2934490326261066	0.5185650137658444	0.4103407860207623
0.3769120600329700	0.6308625215263145	0.6498158485294651
0.3236280954254366	0.6108869148638415	0.3464765209387560
0.2167276819929349	0.6144577766509557	0.3284430540502002
0.5050777329306532	0.5562636122859098	0.3836397665982115
0.1239289358268775	0.4832939050633498	0.4284913034714279
0.3782307559665112	0.6401476302852481	0.5137938827473920
0.5889482857408185	0.5638037166513185	0.4334592892784889
0.7098650374203644	0.5370456146676176	0.3376624960265605
0.3346732496687112	0.4773884869993447	0.3845865224357736
0.3726490724776595	0.5931600069610659	0.5050689987167998
0.5237712059167829	0.5397752699913472	0.3102647929990804
0.3693305453224565	0.5649916340496796	0.5688692315967947
0.6261447677060477	0.5300035282375118	0.2878954158325056
0.6910962425810725	0.5541187124788849	0.4100595466537500
0.3956562466613324	0.6427368760990695	0.3187408101887269
0.3715548115479417	0.5840729843170102	0.6410223548456748
0.2704028612438936	0.4392867733033249	0.3814394283155708
0.1649084425884364	0.4424422997435901	0.4024242382469281
0.3602825756285731	0.6782644916989020	0.2739025027768079
0.1827745642666922	0.6497706665981782	0.2825065976103289
0.2540038893363792	0.6817684183230392	0.2555700339078301
0.3120209509611254	0.4787316801522851	0.9337626915770552
0.1198266368802293	0.3412936813791443	0.0861397684170611
0.2065533725272798	0.4814442337566607	0.9103363359161580
0.1231003864518230	0.3691391849373982	0.1498168227289315

0.1763855658715967	0.3891147950578268	0.8464917346223177
0.2832826665274426	0.3855484308542322	0.8284454419684046
0.9949242180872230	0.4437390241700367	0.8836379139965125
0.3760713587458545	0.5167121768724714	0.9284903661112984
0.1217288708217229	0.3598646263118892	0.0137950341203373
0.9110531958277556	0.4361963095870512	0.9334572690664743
0.7901354732022872	0.4629542482761652	0.8376608626675202
0.1653253899794174	0.5226191269356760	0.8845763978942993
0.1273515290962079	0.4068519684273036	0.0050754627315543
0.9762297020030933	0.4602272910987768	0.8102624700566284
0.1306862831674262	0.4350149475720416	0.0688796563087439
0.8738548452860500	0.4699967672562517	0.7878932139551587
0.8089048692377625	0.4458807851000286	0.9100576535947655
0.1043603340725899	0.3572639954191031	0.8187517403720777
0.1284637847187343	0.4159286827332547	0.1410303203048580
0.2295979863987540	0.5607196696743488	0.8814309775098562
0.3350917122320694	0.5575630514698776	0.9024198523992065
0.1397331947266313	0.3217336667680619	0.7739163519347044
0.3172367062961260	0.3502394889854724	0.7824957619109029
0.2460103056446665	0.3182337054982381	0.7555708295112155
0.8120211270060775	0.0212727282004872	0.5662352398912903
0.6198245502338274	0.1587081479460915	0.4138594061840062
0.7065537840699945	0.0185593319819379	0.5896604221471144
0.6230977629455988	0.1308625532464842	0.3501824381255164
0.6763846206486569	0.1108864368612198	0.6535100508389031
0.7832823578825899	0.1144525727547334	0.6715558189138778
0.4949242360041883	0.0562617987622862	0.6163611465602861
0.8760732908264927	0.9832926491847581	0.5715088357620601
0.6217278366177285	0.1401376803276417	0.4862042375684429
0.4110531643384018	0.0638041062279966	0.5665418812773307
0.2901353426557818	0.0370466901224551	0.6623388758882988
0.6653264776952854	0.9773839365686882	0.6154175323164989
0.6273506763621989	0.0931505705220642	0.4949241231249562
0.4762297860575833	0.0397742457122112	0.6897369504077233
0.6306854601890989	0.0649870814307573	0.4311200550638242
0.3738550996694625	0.0300041068833791	0.7121062300158009
0.3089047431549546	0.0541200291041509	0.5899418793805369
0.6043593690319167	0.1427374740284366	0.6812494065516609
0.6284615763519238	0.0840730832501519	0.3589695762049478
0.7295993264314160	0.9392835645670697	0.6185626813322292
0.8350929622628560	0.9424411879571257	0.5975754272291454
0.6397329855072111	0.1782678281456158	0.7260844346565936
0.8172359225709256	0.1497614805627091	0.7175054642425137
0.7460102389123130	0.1817673963710826	0.7444300711750054

0.6879786000744926	0.9787295318915076	0.0662370050557403
0.8801767027768816	0.8412930084388964	0.9138604588706822
0.7934467534254983	0.9814430077162942	0.0896624845400543
0.8769014517385286	0.8691387338005531	0.8501835916565154
0.8236164612282845	0.8891147352787990	0.1535098710183068
0.7167195146342665	0.8855485827320324	0.1715567484491659
0.0050764855967097	0.9437378374790867	0.1163617668304820
0.6239266383633183	0.0167099292166310	0.0715107276850342
0.8782735926387133	0.8598638372164961	0.9862048646870970
0.0889477711012722	0.9361951294768966	0.0665428071469590
0.2098654683051888	0.9629546560143404	0.1623379853510201
0.8346750034792920	0.0226183541938281	0.1154187483950071
0.8726499218950231	0.9068509688164003	0.9949242299926776
0.0237711098692858	0.9602260320666394	0.1897369722027825
0.8693150912408567	0.9350146330722230	0.9311203909207070
0.1261456665384895	0.9699968624026130	0.2121056142836404
0.1910961770632163	0.9458798627228572	0.0899420392554330
0.8956400195937688	0.8572623568027924	0.1812472962321396
0.8715378260566978	0.9159281942013484	0.8589702379289419
0.7704014111113515	0.0607187081312633	0.1185636689640621
0.6649068340864057	0.0575613293682633	0.0975781854938122
0.8602665679271982	0.8217320706004114	0.2260826321634794
0.6827663455922222	0.8502398556176128	0.2175071966184884
0.7539901264080439	0.8182334059005137	0.2444318617085969
0.8442597228436325	0.4472533203665970	0.5442880224711963
0.6174886453105156	0.3047869972663763	0.4068964037383235
0.6226433364295800	0.3540274720857511	0.2937514675201412
0.8405709958624491	0.4098208520079570	0.6507931183773038
0.9586975445031360	0.5141595230345459	0.5559913333712682
0.6204166854406707	0.3376252958255864	0.5352906285408917
0.4252997535295135	0.4226675837815190	0.5101262080536284
0.2098811631869272	0.4707675996597330	0.6794554688685450
0.5835439321475295	0.5251121168450140	0.6330022172280476
0.5400298730495210	0.4651344252857624	0.7300031037513119
0.6366272134199967	0.4714827092010035	0.4376809403983833
0.3602723948697302	0.4829909072026861	0.7690105704267040
0.2437893552574439	0.4405030999717166	0.5509865256661425
0.5212929269231149	0.3596896909491704	0.6668933953966293
0.6316715128200597	0.4378465462056935	0.3094851265607507
0.6975230705814498	0.5930072012178516	0.6374074581882145
0.8850023089350035	0.5873734577073229	0.6015778803348694
0.5835878655384903	0.2967554872623966	0.7466318521574352
0.8999437638931246	0.3481738108860644	0.7329298453080947
0.7737195877936239	0.2910748319006314	0.7808831982746135

0.6557405878913054	0.5527496925783901	0.0442879794715541
0.8825105336655774	0.6952141056137581	0.9068952002712434
0.8773612914775251	0.6459732486442458	0.7937500424486853
0.6594274365227666	0.5901798645471622	0.1507939871946390
0.5413037167935889	0.4858442819514668	0.0559917510922338
0.8795828446135269	0.6623756194713053	0.0352903999520535
0.0746989442020348	0.5773329672630807	0.0101262838347385
0.2901192916373300	0.5292380856271784	0.1794592362453166
0.9164516650576445	0.4748919328760155	0.1330056448239313
0.9599707297375673	0.5348687852888687	0.2300051485087529
0.8633728571796392	0.5285189517163261	0.9376816078606142
0.1397282266322347	0.5170141223500327	0.2690137125993272
0.2562116654782245	0.5594992963495081	0.0509888717977094
0.9787099617295417	0.6403085543700130	0.1669024472934519
0.8683320208459875	0.5621554033453877	0.8094836585133969
0.8024741423241700	0.4069954669390286	0.1374091666504338
0.6149993006487370	0.4126294074512104	0.1015760984019405
0.9164177739603535	0.7032398083504143	0.2466419214922002
0.6000553256158314	0.6518269434670861	0.2329308875413855
0.7262801042802641	0.7089245559147265	0.2808840493818054
0.1557421865491481	0.9472544416425535	0.4557103822788215
0.3825673416637308	0.8047907302415560	0.5931092217647118
0.3773477157130450	0.8540233134143933	0.7062475343084140
0.1594180239726051	0.9098268621494404	0.3492030398601703
0.0413008562790108	0.0141597016721961	0.4440088546640250
0.3796915115249743	0.8376505914110945	0.4646956716000965
0.5747017599030164	0.9226666215789694	0.4898724402535184
0.7901187721809699	0.9707667422426809	0.3205429657661391
0.4164553277903212	0.0251144511381682	0.3669925027734799
0.4599713721182640	0.9651372922642402	0.2699942121673233
0.3633522709619245	0.9714871169754551	0.5623324157537367
0.6397284354740010	0.9829900699547965	0.2309875801519162
0.7562097226589978	0.9405012465786909	0.4490118449409752
0.4786941059475239	0.8596876501181102	0.3331120475460139
0.3683161545567389	0.9378441091356471	0.6905254624289414
0.3024705510268846	0.0930104572498167	0.3625889831283008
0.1149992245408541	0.0873749779473980	0.3984231094265240
0.4163933748162343	0.7967497574998269	0.2533847223890899
0.1000529134735690	0.8481946216256133	0.2670299388913981
0.2262704335486180	0.7910755289680321	0.2191155578626757
0.3442594489148460	0.0527520639660054	0.9557138516683182
0.1174874895767512	0.1952146870693307	0.0931047719530432
0.1226410676843605	0.1459743931707294	0.2062492187089097
0.3405710075772375	0.0901807867989352	0.8492069185432728

0.4586980493747576	0.9858461002092711	0.9440080156112448
0.1204162431691448	0.1623774695207310	0.9647111092804468
0.9253004046838157	0.0773348061956736	0.9898731930641368
0.7098804775917471	0.0292353833103147	0.8205437192470003
0.0835477608759076	0.9748925563051383	0.8670018060543263
0.0400293199239588	0.0348693713301100	0.7699963014726577
0.1366271702630489	0.0285194193107449	0.0623176875104979
0.8602727084916054	0.0170116495273082	0.7309887841924864
0.7437879567256189	0.0595002087436081	0.9490115964058523
0.0212924809834389	0.1403132526706446	0.8331074711738702
0.1316699363985831	0.0621558558289110	0.1905158937796828
0.1975276697681125	0.9069948102965371	0.8626032671218780
0.3850007546426615	0.9126305935099257	0.8984253989001909
0.0835894798886222	0.2032472342593220	0.7533699164294785
0.3999442119461468	0.1518276514589427	0.7670692286231126
0.2737196579761586	0.2089272463765203	0.7191178345412189
0.1557379487045097	0.5527498863116528	0.4557104640489816
0.3825099194817136	0.6952139397758403	0.5931048427089761
0.3773611094737641	0.6459732173085506	0.7062499278081026
0.1594272482956035	0.5901803185112342	0.3492070515251794
0.0413000446926477	0.4858441502565100	0.4440061168991545
0.3795825890391156	0.6623754198219984	0.4647096436313389
0.5747004279998069	0.5773325921257243	0.4898752886996453
0.7901190766897636	0.5292357694101597	0.3205431816788059
0.4164546635744146	0.4748911023283732	0.3670048356002982
0.4599715064838159	0.5348714045354035	0.2699953596694262
0.3633736740337101	0.5285187654399535	0.5623185654752882
0.6397286867287867	0.5170144359405603	0.2309876047355536
0.7562113647628541	0.5594978591021703	0.4490132902760777
0.4787096711227843	0.6403084549168179	0.3330977095785256
0.3683329919685052	0.5621550658778364	0.6905158699867833
0.3024759504153166	0.4069939830430892	0.3626054982042415
0.1149964980774427	0.4126294207329369	0.3984248404175640
0.4164177173013581	0.7032398305323919	0.2533580054027891
0.1000550644525031	0.6518271508850557	0.2670697962555492
0.2262800503166085	0.7089245898661014	0.2191157360312737
0.3442612624682940	0.4472561097651228	0.9557076625493993
0.1174352519601627	0.3047913132319679	0.0931090715147608
0.1226504718704352	0.3540244672859083	0.2062480724774902
0.3405841159378893	0.4098274360505685	0.8492031744987285
0.4587001377274884	0.5141616572611616	0.9440074790674479
0.1203099226808796	0.3376527529773621	0.9646942311221003
0.9252977109220050	0.4226688564125478	0.9898745211980085
0.7098820390694492	0.4707663774908274	0.8205424106961036

0.0835432874338899	0.5251155796043873	0.8669958043018958
0.0400281659097468	0.4651352750899808	0.7699932374135116
0.1366472317016487	0.4714879660705220	0.0623334121459203
0.8602705265275028	0.4829872801442186	0.7309863850236163
0.7437911528580271	0.4405035474022462	0.9490135026498605
0.0213042321919246	0.3596924093900686	0.8331022956319071
0.1316812475978316	0.4378448793701273	0.1905256730888255
0.1975259756787079	0.5930110917717406	0.8625910950636967
0.3850038758556766	0.5873760504392516	0.8984225479678566
0.0836002130903911	0.2967571566753612	0.7533729877272579
0.3999487667495524	0.3481954869682403	0.7670296476798495
0.2737304295911674	0.2910782024917555	0.7191135217509893
0.8442597843546561	0.0527485869440234	0.5442913117558809
0.6174340695751389	0.1952104587022798	0.4068897050269324
0.6226481946234410	0.1459774397849913	0.2937511302789588
0.8405841178935429	0.0901742604875811	0.6507968361892646
0.9587018304108185	0.9858439868581880	0.5559937151663946
0.6203095775165172	0.1623502157749475	0.5353042983220297
0.4252983269481723	0.0773335711637156	0.5101260321986676
0.2098813211775698	0.0292366291305224	0.6794583596503658
0.5835453664348081	0.9748884612135909	0.6330010001354857
0.5400275590608402	0.0348685234851585	0.7300073130742214
0.6366471693454268	0.0285145295815028	0.4376680887751305
0.3602707933242210	0.0170152720910974	0.7690142252909935
0.2437895546084827	0.0594997977122884	0.5509883174804948
0.5213036018899381	0.1403105360094481	0.6668968293614742
0.6316796934620332	0.0621575124574792	0.3094734884020739
0.6975308010373451	0.9069903029171456	0.6373982957673934
0.8850022003737772	0.9126277824860625	0.6015741617781732
0.5836013862056102	0.2032455797431445	0.7466253988905356
0.8999486293722041	0.1518063168132082	0.7329704411471563
0.7737305034901116	0.2089239512019733	0.7808853857410950
0.6557399834564065	0.9472542076547785	0.0442911524224560
0.8825671123982867	0.8047907435271897	0.9068908793045999
0.8773475359748028	0.8540233374728019	0.7937526476365900
0.6594178436130972	0.9098263938384533	0.1507958427676015
0.5412984727743403	0.0141597263987726	0.0559929327149798
0.8796912312226435	0.8376507874043787	0.0353044581552827
0.0747032392066830	0.9226670096066938	0.0101260201264468
0.2901185443871080	0.9707690726302128	0.1794546416854911
0.9164581688035197	0.0251152795032342	0.1329970973144445
0.9599721380136743	0.9651346955447097	0.2300052874744053
0.8633531219944820	0.9714873044237193	0.9376674098850469
0.1397288822522764	0.9829897512484759	0.2690111398351473

0.2562096810232639	0.9405026134915102	0.0509860380499697
0.9786938160263599	0.8596877445369533	0.1668877797878500
0.8683170468964397	0.9378444110890869	0.8094750201825412
0.8024722398958180	0.0930120242077287	0.1373966038314566
0.6149964186587448	0.0873748359093813	0.1015759441218111
0.9163932923544124	0.7967497276667956	0.2466153362375378
0.6000526423141262	0.8481944294118424	0.2329693865466265
0.7262703622760297	0.7910755112191687	0.2808846584718047
0.6273780482628240	0.4309884690326887	0.5883760381706425
0.8726226833975610	0.5690141445024487	0.0883767658591732
0.3726174662128947	0.9310001559725855	0.4116286447454475
0.1273778245854288	0.0690155451455041	0.9116244611084933
0.3726230187046366	0.5690139848497128	0.4116237051331982
0.1273821137284928	0.4310016381307939	0.9116277780767439
0.6273805388352093	0.0690033042422365	0.5883712113753398
0.8726179241336016	0.9310002003295609	0.0883701138857589

1-CN in POSCAR format

Note that the fragmentation we used comprised a single HAT molecule in the first fragment and the rest of the system in the second fragment. The first fragment corresponds to the first thirty atoms in the following structure.

```
N C N C N C Pt N C H C H P
   1.0000000000000000
   12.7314000000000007    0.0000000000000000    0.0000000000000000
    0.0000000000000000   30.6722999999999999    0.0000000000000000
    0.0000000000000000    0.0000000000000000   17.5125999999999991
  N   C   N   C   N   C   Pt  N   C   H   C   H   P
   12   18   36   54   16   16    4   12   24   36  192  160    8
Direct
 0.1199151087006104  0.7039825805330348  0.9113406590517207
 0.1194885251297641  0.6592735709312704  0.7750265763002223
 0.1394805313006084  0.7056643670827065  0.6368321060453407
 0.1145845788815087  0.6837209047206041  0.1033663526469532
 0.1223206182038743  0.6237734679175532  0.5064848710110915
 0.0927808407620392  0.5550417821884892  0.7041885228791475
 0.1199032353700880  0.7960970786200441  0.9113988308614807
 0.1194703229283232  0.8408720407699507  0.7751605042120326
 0.1392241367813191  0.7945594060649436  0.6368820418927362
 0.1145247557521102  0.8162634596874189  0.1034614526059037
 0.1219384638160501  0.8766758745976175  0.5068157038262019
 0.0927633861284751  0.9451424298722412  0.7044543479607036
 0.1171058430085852  0.7268332901316619  0.9758140731178440
 0.1185801522672394  0.6391250837603605  0.7078367455483444
 0.1277012534022003  0.7030394522077287  0.7740676732868499
 0.1246783135252757  0.7268258273322060  0.8460540373621704
 0.1297043049069085  0.6626168462078526  0.6380724305788354
 0.1365026128651550  0.7263365058546468  0.7043811068361180
 0.1148907836717906  0.7029750352131968  0.0459152838357451
 0.1043739679241050  0.5927844640833685  0.7068810151232297
 0.1265042553351526  0.6409231402875194  0.5658565891958380
 0.1170958135120120  0.7732179701662085  0.9758420612602773
 0.1185182637128328  0.8610575346100379  0.7080117095423122
 0.1276840042432011  0.7971070810417983  0.7741254273841751
 0.1246755639252206  0.7732827405924735  0.8460819545268012
 0.1294864477012996  0.8376133188439141  0.6381864039959466
 0.1364389718702896  0.7738374870486683  0.7044027035658474
 0.1148525628543845  0.7970429527378691  0.0459762858683115
```

0.1043530214132601	0.9073990087697131	0.7071141565131633
0.1261878390391448	0.8593837705392969	0.5660532592375548
0.3800967057385179	0.2960971340491599	0.4113988053287863
0.3805296664375741	0.3408721131615570	0.2751604378986307
0.3607758664959785	0.2945594805581161	0.1368820059122490
0.3854753425770765	0.3162633952918226	0.6034614843121592
0.3780616980579212	0.3766759098784833	0.0068155770899893
0.4072366510523727	0.4451425213568704	0.2044545296728157
0.8800843045849144	0.2039829087661911	0.0886593599009704
0.8805114842355787	0.1592736856485932	0.2249736484326768
0.8605196261476503	0.2056642521817506	0.3631677217089925
0.8854153863285054	0.1837213784759829	0.8966336624147893
0.8776796963898605	0.1237738335474655	0.4935155284743959
0.9072189968947709	0.0550418917734586	0.2958113981733481
0.6199033311113826	0.7960974076500238	0.5886012090186098
0.6194703358045464	0.8408721580032796	0.7248398394647579
0.6392235463782852	0.7945594941799624	0.8631180265107709
0.6145247060880180	0.8162633950802045	0.3965383883330901
0.6219382140731196	0.8766758575984268	0.9931843867455858
0.5927632103918348	0.9451424459865105	0.7955455065002841
0.6199156589423961	0.7039828979768393	0.5886593384990892
0.6194885164102479	0.6592736277634139	0.7249735368926408
0.6394803886940771	0.7056642646005814	0.8631678051573813
0.6145848876426543	0.6837214196088958	0.3966335928552531
0.6223202870975876	0.6237734391842283	0.9935153173355408
0.5927808643796041	0.5550417863393489	0.7958113126866065
0.8800959875947302	0.2960974450537135	0.0886012072264679
0.8805296030416304	0.3408721778755929	0.2248398715060485
0.8607759190390087	0.2945594854820609	0.3631180052852778
0.8854753875927391	0.3162633956118998	0.8965383626200167
0.8780617630998272	0.3766758466879286	0.4931847214404804
0.9072366703734731	0.4451424683389913	0.2955456295715199
0.3800843507599985	0.2039828598105149	0.4113406651248321
0.3805114770520209	0.1592736573917522	0.2750264008611738
0.3605195420254895	0.2056642649490331	0.1368322533275605
0.3854155076383790	0.1837214140664680	0.6033664567032598
0.3776796703152359	0.1237734623409934	0.0064847966319049
0.4072191199936389	0.0550418697373724	0.2041885736955140
0.3829041187867183	0.2732180098458488	0.4758419492509964
0.3814816091480083	0.3610576545911653	0.2080116208917829
0.3723160652470144	0.2971071742056922	0.2741253288316638
0.3753245170794183	0.2732828249762678	0.3460819529872301
0.3705134432860810	0.3376134319763717	0.1381863312920116
0.3635611095123191	0.2738375536890739	0.2044026304632644

0.3851473915470182	0.2970429773277045	0.5459762524976209
0.3956467865579534	0.4073991089517575	0.2071141472625760
0.3738120872052422	0.3593838554214832	0.0660531623680759
0.8828939803936653	0.2268333959994224	0.0241862086936100
0.8814200541527158	0.1391252708756671	0.2921631472396525
0.8722986681980178	0.2030396557017768	0.2259326287022873
0.8753214047655049	0.2268259975687349	0.1539458356104213
0.8702959364131917	0.1626172171401792	0.3619277781661714
0.8634974154527096	0.2263365567130979	0.2956188957792852
0.8851090558984367	0.2029752580847259	0.9540848063753621
0.8956260872313454	0.0927845771142999	0.2931189764502118
0.8734960875685402	0.1409233873320864	0.4341438295655563
0.6170960575714607	0.7732180712276647	0.5241577746383003
0.6185184925804192	0.8610575751620366	0.7919886176829279
0.6276841193321047	0.7971072216896951	0.7258747962199061
0.6246756575063461	0.7732828415601865	0.6539183182046446
0.6294864266499979	0.8376134384447113	0.8618136531030026
0.6364388653315437	0.7738375754850163	0.7955974691594864
0.6148527749295710	0.7970430531383217	0.4540236363163574
0.6043530846666959	0.9073991329659075	0.7928860801805102
0.6261876555764840	0.8593838400748995	0.9339469720162417
0.6171062263682454	0.7268333836193650	0.5241861792136681
0.6185799515348458	0.6391252140086343	0.7921627765694579
0.6277011032286592	0.7030396069260401	0.7259323711660104
0.6246783657530933	0.7268259183332082	0.6539458409495458
0.6297040397282863	0.6626171997293430	0.8619276838793576
0.6365023352913983	0.7263365791004375	0.7956188780205748
0.6148912870241383	0.7029752498565176	0.4540847149822261
0.6043739445158083	0.5927845706133865	0.7931187563329036
0.6265039223152979	0.6409232335742336	0.9341435455694101
0.8829039880696116	0.2732180856426886	0.0241578030472734
0.8814815590172567	0.3610575885671496	0.2919886299947311
0.8723155875918974	0.2971072364658073	0.2258748159366876
0.8753239485243185	0.2732828850933455	0.1539183241942020
0.8705134879661822	0.3376134483966226	0.3618136675124289
0.8635608594477140	0.2738375897012648	0.2955974668200154
0.8851474210641123	0.2970430398659071	0.9540236982222924
0.8956469367594816	0.4073990902720878	0.2928861397722075
0.8738122854480248	0.3593838050826886	0.4339469306658748
0.3828939949577244	0.2268333525648950	0.4758137520826454
0.3814197907834365	0.1391252215455336	0.2078368683742218
0.3722987026389504	0.2030396013378343	0.2740673631036593
0.3753216582368042	0.2268259574012945	0.3460542527872539
0.3702955917955907	0.1626171861937732	0.1380723325149810

0.3634974635776972	0.2263365471219184	0.2043811348999999
0.3851091252344859	0.2029752076211608	0.5459152718711067
0.3956259296148354	0.0927845455318023	0.2068809880217876
0.3734957860792216	0.1409232351007546	0.0658563554745757
0.8860451460782133	0.6473642225008763	0.6469540395819290
0.6140612566490599	0.3526880059026413	0.1470434183596996
0.8892200881997557	0.7500221364001435	0.8201772487687699
0.8724234280219917	0.7499998601154472	0.4598000065894687
0.1139543228972607	0.1473643285512682	0.3530468536547837
0.3859386760439741	0.8526881940978924	0.8529546904262780
0.1107783382122595	0.2500221326494876	0.1798227577705192
0.1275777434765041	0.2500000626277519	0.5401999460798821
0.3860447549421164	0.6473643104054982	0.8530468861962625
0.1140611615673386	0.3526881859530654	0.3529551929795059
0.3892216110170559	0.7500221419662451	0.6798227302949144
0.3724223388011785	0.7500000799778879	0.0401999387881814
0.6139540999885307	0.1473641465056008	0.1469539580032503
0.8859377712732157	0.8526881248929860	0.6470441754206464
0.6107789638161538	0.2500219081027195	0.3201767708046512
0.6275778964217746	0.2499998760286157	0.9597997133483965
0.8807614065987206	0.6855394190432671	0.6424498661570424
0.6193098066583138	0.3145133964011022	0.1424881010896570
0.8755071305231872	0.7500163531551570	0.5269473241404901
0.8840361013685035	0.7500224746308398	0.7531405998222698
0.1192385323857536	0.1855395209361849	0.3575503129386535
0.3806900675728901	0.8145136508826170	0.8575109643226981
0.1244935387434356	0.2500165774510722	0.4730526191640541
0.1159631253346571	0.2500224662807295	0.2468594468854103
0.3807609281535559	0.6855395096367817	0.8575504536471067
0.1193097146180557	0.3145136205155548	0.3575109976906309
0.3755063569406966	0.7500165941499347	0.9730525948409140
0.3840366500139938	0.7500224599219277	0.7468593067097089
0.6192382597549168	0.1855392998666658	0.1424496867631597
0.8806897127020418	0.8145135758580687	0.6424885151522303
0.6244935796370116	0.2500162806821561	0.0269470068153977
0.6159632574263889	0.2500222838453726	0.2531401992823276
0.8787195903279271	0.7500271829006763	0.6402839951535733
0.1212803030217448	0.2500273531184417	0.3597160095016906
0.3787203084219670	0.7500273668623556	0.8597159048077827
0.6212801520769360	0.2500269987480242	0.1402836465627741
0.9669053331434022	0.5776712866805567	0.3620236744817407
0.5331712703047963	0.4223379096011163	0.8619133765188202
0.8816054488506937	0.7499579071759543	0.0441537361022557
0.0330945855450121	0.0776718232311273	0.6379770429014474

0.4668288304965150	0.9223379157810410	0.1380864885256518
0.1183945203271705	0.2499578565653943	0.9558462813324092
0.4669052689745486	0.5776713301487612	0.1379759763528749
0.0331711685310625	0.4223379272445862	0.6380866825805789
0.3816057844149292	0.7499578993268082	0.4558452005389937
0.5330944890535365	0.0776711966052961	0.8620245982239202
0.9668288952901709	0.9223378428686586	0.3619124765521327
0.6183945149521447	0.2499578727695574	0.5441547157196601
0.9260904492997188	0.6056734861542750	0.3960336005976790
0.8737661390861844	0.6400098029906599	0.4379338162575648
0.5738621720799516	0.3943316286145730	0.8959904211180021
0.6260422688584084	0.3599880859576359	0.9379668181323348
0.7900221868456376	0.7497052009347147	0.0468743473692418
0.6762586347157695	0.7494566287055336	0.0491894195515463
0.0739099601358639	0.1056737206797059	0.6039667357905387
0.1262347112121850	0.1400098478609748	0.5620662528459215
0.4261376806480652	0.8943316551660448	0.1040094599737412
0.3739571503412830	0.8599880818645624	0.0620331426066892
0.2099777444078821	0.2497052056248366	0.9531256393927658
0.3237413098672292	0.2494567361070588	0.9508105210212084
0.4260902867535096	0.6056735644230614	0.1039661608235271
0.3737658686753863	0.6400098630500891	0.0620660067260382
0.0738623458321516	0.3943317251273247	0.6040097321090725
0.1260427376579653	0.3599881675400396	0.5620332739330228
0.2900223616114913	0.7497051630553732	0.4531248985359888
0.1762586810020201	0.7494566182647411	0.4508100083624320
0.5739095111306792	0.1056733636094042	0.8960340510922857
0.6262341030214326	0.1400097272454337	0.9379339112703619
0.9261379614761930	0.8943316889207756	0.3959898447459652
0.8739576789346627	0.8599882237847770	0.4379666328893066
0.7099777692048140	0.2497052455331608	0.5468750034449021
0.8237413623467421	0.2494567437858967	0.5491900628152658
0.9083663610774448	0.6720459442824841	0.4252553122716875
0.7904025099048201	0.6410965975486104	0.4217438082135876
0.8799223138635935	0.6344793746152134	0.4998468703335880
0.6478727417929003	0.7177345163036909	0.0698889416868064
0.6477567012223535	0.7751954810234678	0.0876479877572971
0.6450488470772781	0.7550246342585141	0.9913894733551514
0.5911895141963744	0.3279790304838653	0.9254506746941976
0.7093689343765883	0.3587881174021790	0.9217041936165006
0.6200321575996596	0.3656025025067774	0.9998637018744930
0.0916347025830059	0.1720461073342723	0.5747446695103201
0.2095983091088512	0.1410965274811578	0.5782561574841550
0.1200782455291218	0.1344793393277493	0.5001532303374531

0.3521274493224937	0.2177347211880149	0.9301110327583973
0.3522429522565899	0.2751956602041202	0.9123518213651348
0.3549511073348234	0.2550248794754313	0.0086103856319576
0.4088098248456785	0.8279790027007349	0.0745493969812181
0.2906306370431891	0.8587882690158839	0.0782958436268957
0.3799673304735879	0.8656024556105033	0.0001362133998788
0.4083661012402997	0.6720460839284534	0.0747446083444903
0.2904021687517162	0.6410966891855132	0.0782561346522842
0.3799219869739445	0.6344795096584308	0.0001529629606177
0.1478726284298138	0.7177345384444135	0.4301107568221605
0.1477566293839372	0.7751954601716170	0.4123517967119694
0.1450493090402438	0.7550246163765959	0.5086103147687081
0.0911900694054236	0.3279791601511675	0.5745493979554634
0.2093692073036204	0.3587882771237116	0.5782959106901076
0.1200325598316179	0.3656026339218508	0.5001365078083343
0.5916342390602710	0.1720458514474918	0.9252552935521922
0.7095978342007234	0.1410963812069000	0.9217438391597539
0.6200781343793160	0.1344794380540840	0.9998470693182435
0.8521275221681354	0.2177347694692347	0.5698892795411835
0.8522432231350119	0.2751956941227443	0.5876483482254128
0.8549509469074414	0.2550248638370439	0.4913897680494032
0.9088101259262051	0.8279791890736847	0.4254507343601060
0.7906309725752796	0.8587883906328194	0.4217040523581245
0.8799679039528161	0.8656028724266108	0.4998634194896599
0.6156243195639102	0.3697367708280614	0.3517548399551838
0.6230821865421240	0.4334119564617935	0.4332644071158865
0.6211268788330221	0.4149843475282083	0.3604523757011729
0.6192674227666972	0.4061668494004787	0.4976789252597813
0.6119230971758423	0.3426913340606901	0.4159280204478073
0.6134991549629307	0.3607592277299140	0.4889283002921009
0.8086794050039082	0.4718343311576972	0.5685377016775983
0.7040470188222762	0.4768621804259111	0.5929178463330239
0.7382328666283552	0.5525335999849685	0.6230679457702900
0.8778374476206087	0.5069041432220822	0.5738914596274070
0.6686685081802635	0.5174076793874388	0.6199365923988972
0.4675428353993845	0.4605497064539201	0.6917251627739380
0.2835335484190747	0.4682024668840180	0.6594821658656440
0.7695367036965649	0.3840194357130072	0.6782375146707444
0.3655862362458989	0.4723330079823204	0.7120060537712413
0.6633552552656520	0.3889620798132120	0.6586123103332894
0.4875650719496512	0.4448658317671145	0.6176722140209675
0.8429015156790250	0.5471944619583040	0.6010057157129810
0.5881203771242127	0.3593142013473787	0.6858390953843034
0.7245984688858111	0.3197818654515595	0.7520028903853949

0.4052055736300000	0.4403315470370566	0.5650370400316752
0.6191603328186562	0.3246325750072382	0.7319648028639002
0.3034690474498850	0.4520495002124262	0.5863334525622758
0.7991905744933685	0.3495039247853412	0.7252827154293825
0.8844637073648034	0.6302603378671453	0.8517249640790351
0.8769350892718232	0.5665920014378695	0.9332479104326546
0.8788953030580985	0.5850149993345573	0.8604325813160478
0.8808293353590392	0.5938394619854911	0.9976573520786431
0.8882475325068936	0.6573078669258687	0.9158912521189543
0.8866897298250198	0.6392438044284587	0.9888949149499895
0.6913200488149425	0.5282132872027385	0.0684600557893900
0.7959414601027592	0.5231680363313567	0.0928539480313535
0.7616891657008300	0.4474978944632074	0.1229819014236402
0.6221338418633101	0.4931520005354685	0.0738002892466414
0.8312808929340044	0.4826145856983916	0.1198628430750000
0.0324864349196945	0.5394480285628962	0.1917183359796121
0.2164975478343744	0.5317920079894295	0.1594854402662308
0.7305436891331266	0.6159982683161535	0.1782184020754091
0.1344414628639945	0.5276688907894328	0.2120088441616097
0.8367339089861379	0.6110380121832267	0.1586153264246180
0.0124692867633541	0.5551211567089459	0.1176585922390518
0.6570308993305226	0.4528550427238575	0.1009138240724917
0.9119962613824310	0.6406556451206777	0.1859037409675279
0.7755272550627935	0.6801996297323967	0.2520541859910910
0.0948296922240841	0.5596456979944088	0.0650229696342938
0.8809760438725820	0.6753226593716330	0.2320725972288040
0.1965650049498899	0.5479304246128415	0.0863264429767451
0.7009067571107812	0.6505111231419090	0.2252701005474791
0.3843756483556200	0.8697365210679996	0.6482448871675607
0.3769179168320427	0.9334120536958344	0.5667351802215347
0.3788731635132974	0.9149844271783799	0.6395473879736102
0.3807326131589929	0.9061671843002915	0.5023208790901594
0.3880767932644024	0.8426912502986591	0.5840715568565216
0.3865008052037675	0.8607591341969328	0.5110715785123436
0.1913206559737501	0.9718343307673696	0.4314622101165021
0.2959522961090283	0.9768622095488330	0.4070822656604928
0.2617672011440910	0.0525336888013393	0.3769320694093078
0.1221623085502382	0.0069042205628196	0.4261085356206873
0.3313311894117992	0.0174081855252926	0.3800633044962231
0.5324570594205037	0.9605498496073820	0.3082746573703245
0.7164665113804477	0.9682024787882185	0.3405176549832094
0.2304633303980731	0.8840194454916011	0.3217624018428644
0.6344136762369692	0.9723331242702299	0.2879936376904957
0.3366447576289637	0.8889621800322760	0.3413875492237680

0.5124349372537953	0.9448659292331670	0.3823273208153943
0.1570978685252602	0.0471944253663240	0.3989944697753555
0.4118798827480430	0.8593143647100140	0.3141607657941370
0.2754017526570262	0.8197818919845632	0.2479969772988384
0.5947945054558361	0.9403315251461963	0.4349626131987904
0.3808402691820665	0.8246327010237865	0.2680351815659781
0.6965311294865566	0.9520494716450144	0.4136660519906447
0.2008095550960962	0.8495038903402737	0.2747171919679918
0.1155359012350479	0.1302603900973481	0.1482751423635756
0.1230648532841019	0.0665920387537847	0.0667521716896534
0.1211044459870338	0.0850149878395144	0.1395674456771422
0.1191705321182937	0.0938395612234231	0.0023425901109221
0.1117518121841720	0.1573078691784769	0.0841092829024052
0.1133097647984463	0.1392437938103090	0.0111051390474930
0.3086800341118540	0.0282133411762040	0.9315402133358913
0.2040587036598605	0.0231680634958746	0.9071460759300156
0.2383111675380155	0.9474979521493920	0.8770180211214708
0.3778660983751030	0.9931520862305347	0.9262000718395677
0.1687191541491622	0.9826146891881021	0.8801370666231070
0.9675136086580015	0.0394481521037300	0.8082816300749085
0.7835025462658165	0.0317919894233295	0.8405145948014471
0.2694563125229509	0.1159984592758476	0.8217816539928753
0.8655586326646956	0.0276689477193498	0.7879911507247006
0.1632661232780559	0.1110384187782828	0.8413848348164700
0.9875306145506920	0.0551213125837011	0.8823414187278401
0.3429689626406437	0.9528550808018892	0.8990861730609755
0.0880042858175873	0.1406557723008401	0.8140965230838627
0.2244732270116891	0.1801997205132479	0.7479459824419602
0.9051703040819400	0.0596457331859978	0.9349770746181282
0.1190244957951476	0.1753227727367176	0.7679275858686375
0.8034350397553368	0.0479303830862636	0.9136736010609763
0.2990933847484659	0.1505112388009743	0.7747299707483161
0.1156242861424895	0.3697365019639980	0.1482449214412251
0.1230820087002129	0.4334120589854940	0.0667352500055482
0.1211267590216974	0.4149844199020040	0.1395474299645169
0.1192673565144400	0.4061672000734802	0.0023209217586677
0.1119231165678612	0.3426912535676231	0.0840716188340364
0.1134991200684920	0.3607591475316969	0.0110716099490769
0.3086794140687973	0.4718343732592784	0.9314623848828301
0.2040472903536838	0.4768622422373674	0.9070822811727797
0.2382328212062682	0.5525336958500939	0.8769320643120651
0.3778375746012161	0.5069042452578070	0.9261086478239184
0.1686687062017527	0.5174081458043500	0.8800633023244648
0.9675429259887395	0.4605498433079329	0.8082746500156834

0.7835334544584305	0.4682024899367009	0.8405176450280220
0.2695367906378400	0.3840194539811640	0.8217624821915844
0.8655862816275629	0.4723331285876863	0.7879936377401141
0.1633552834156304	0.3889621919635138	0.8413876103426599
0.9875650667775178	0.4448659378997065	0.8823273112610305
0.3429017404479643	0.5471945038724280	0.8989944321354624
0.0881203254542484	0.3593143920242960	0.8141608332690864
0.2245983370303956	0.3197819015366945	0.7479970962165182
0.9052054759188387	0.4403315200996150	0.9349625913356240
0.1191601884063131	0.3246326763161688	0.7680351922511072
0.8034688511755335	0.4520494618341702	0.9136660353091877
0.2991905444251533	0.3495039123762174	0.7747173005743089
0.3844639380006837	0.6302603299512497	0.6482752123236236
0.3769352424623400	0.5665920795400741	0.5667521625342137
0.3788955535254351	0.5850149569585932	0.6395674464145918
0.3808295178826744	0.5938395533541528	0.5023429627086698
0.3882478494503740	0.6573078339569395	0.5841093837943863
0.3866900793554185	0.6392437740724047	0.5111053495541500
0.1913199432824480	0.5282133551693724	0.4315399135797392
0.2959413983276483	0.5231680898502473	0.4071461364223257
0.2616894437227908	0.4474979194141115	0.3770181700635357
0.1221338232591955	0.4931520470068985	0.4261995740590995
0.3312812769981228	0.4826146337888321	0.3801373107260272
0.5324865487450445	0.5394480912472495	0.3082816232768560
0.7164976144687210	0.5317920345875348	0.3405147479607432
0.2305438780309280	0.6159984510222569	0.3217817155192363
0.6344416144618029	0.5276689349259485	0.2879912359649089
0.3367339980237007	0.6110384042997149	0.3413848363230634
0.5124693629608723	0.5551212820972756	0.3823417647797191
0.1570310715144085	0.4528550789691764	0.3990860913355128
0.4119960019238031	0.6406557539217848	0.3140963984225159
0.2755268960349652	0.6801996321789581	0.2479457485290924
0.5948299626705135	0.5596457124178351	0.4349774734665914
0.3809759876961163	0.6753226654410632	0.2679274062106078
0.6965650110848177	0.5479304164360679	0.4136738820879388
0.2009067516419382	0.6505112046257555	0.2747299024179061
0.8843754688166916	0.8697363856000138	0.8517551120439643
0.8769178475705525	0.9334119807017943	0.9332644763067723
0.8788730507545659	0.9149843810537460	0.8604524833985939
0.8807326040983295	0.9061668453593308	0.9976790453185456
0.8880764728079994	0.8426912517510656	0.9159284111059380
0.8865006325830691	0.8607592425898807	0.9889284265505751
0.6913206739077205	0.9718343089192307	0.0685376110982507
0.7959527408295324	0.9768622055364693	0.0929177484341951

0.7617671434474803	0.0525336304948659	0.1230679964575693
0.6221624520155246	0.0069041649552682	0.0738913824849663
0.8313312942015392	0.0174081116412027	0.1199367750895891
0.0324572487087566	0.9605498546061629	0.1917252476356331
0.2164668043633368	0.9682025007756844	0.1594821605139191
0.7304633831382416	0.8840194943492684	0.1782375481247504
0.1344138951595392	0.9723331270975620	0.2120061562347825
0.8366448104651489	0.8889621659954023	0.1586123904980160
0.0124350369220000	0.9448658933645260	0.1176722883911136
0.6570982237719182	0.0471944084208276	0.1010056289968766
0.9118798789142709	0.8593143014538728	0.1858392064596580
0.7754016419365245	0.8197819465151271	0.2520030430559509
0.0947944868320847	0.9403315355665216	0.0650371326843181
0.8808401820268901	0.8246327103196617	0.2319648547472492
0.1965311513058502	0.9520494788218806	0.0863334959581584
0.7008095156037557	0.8495039570392325	0.2252827773031864
0.6155360223277266	0.1302603777938515	0.3517249369623497
0.6230647499601695	0.0665920291697732	0.4332479374860315
0.6211043522446260	0.0850149797780847	0.3604326286180460
0.6191705321215977	0.0938395465677237	0.4976575007286085
0.6117522157251329	0.1573078640158289	0.4158907860557665
0.6133100417322466	0.1392437861554176	0.4888949358184702
0.8086801806705533	0.0282133823847966	0.5684601294812132
0.7040587442393673	0.0231680724163530	0.5928542292140513
0.7383111893005545	0.9474979673765608	0.6229819267009128
0.8778662168851958	0.9931520889110593	0.5738002998909378
0.6687192135569406	0.9826147154583158	0.6198629466750513
0.4675135498144721	0.0394481043172210	0.6917184898291860
0.2835024650388797	0.0317919926739425	0.6594854659424179
0.7694561729866441	0.1159984557336631	0.6782184170964289
0.3655585584448090	0.0276689421040705	0.7120089583710729
0.6632660307154915	0.1110383964592167	0.6586152476367415
0.4875307150044056	0.0551212936757723	0.6176582379918804
0.8429689967058229	0.9528551118533172	0.6009138833452039
0.5880040247924188	0.1406557489456302	0.6859036451525928
0.7244730788426924	0.1801996697775954	0.7520542755080722
0.4051703799697463	0.0596456696112934	0.5650226564887002
0.6190240095740097	0.1753227043643463	0.7320725786045684
0.3034349805851750	0.0479303360000862	0.5863263286922937
0.7990932520824954	0.1505112167387844	0.7252701747620811
0.6150641043966379	0.3555766494582428	0.2945240643009522
0.6292163298311373	0.4686714661532818	0.4398817765561070
0.6246209233688038	0.4360712353153409	0.3103647739436607
0.6095380623357166	0.3073866436507491	0.4085507936831941

0.6118162495447398	0.3393794646225945	0.5386328382737449
0.8365297558480300	0.4407051597351740	0.5462602833208016
0.7114384964373980	0.5843759065542208	0.6429832882296017
0.9599085609469381	0.5023236555121130	0.5581171162501528
0.5876435570864658	0.5216170669426941	0.6385967602370294
0.5298750338623900	0.4628860455679271	0.7342183054815907
0.2037725119421112	0.4775618506541668	0.6752354956517052
0.8293669280777068	0.4065569143629119	0.6577568739365836
0.3507430368721579	0.4845982292176942	0.7694458864956782
0.8960204265674908	0.5749150823610326	0.6060228248578083
0.5057762047352595	0.3629270502761768	0.6701552213923044
0.7486946839110191	0.2932783088398722	0.7896232060551033
0.4203070623251186	0.4275833203992203	0.5079716843640564
0.5606040925668171	0.3014378130511970	0.7524686705134215
0.2396645273974792	0.4488935825604585	0.5452222316247438
0.8812774822522869	0.3461190794955650	0.7417618052869718
0.8850096001134063	0.6444178591921099	0.7944927546547754
0.8707346365933699	0.5313350729798809	0.9398732541278633
0.8753420526800820	0.5639263851276489	0.8103493413403786
0.8906520760043396	0.6926117810634964	0.9085059201296473
0.8884615330096849	0.6606203796341837	0.0386041075451084
0.6635014562138922	0.5593510350811310	0.0461950189577316
0.7884519632004029	0.4156499423477362	0.1428908188942917
0.5400674223623642	0.4977433291045495	0.0580209875845627
0.9122988903366600	0.4783953250055438	0.1385319832391501
0.9701486591305987	0.5371151397152839	0.2342092212639548
0.2962575013356491	0.5224367819191190	0.1752483459889961
0.6707008120734099	0.5934758742184609	0.1577086078386881
0.1492833021047089	0.5154134582474641	0.2694553661542125
0.6038831806120497	0.4251440505807343	0.1059235482425776
0.9943456691415206	0.6370281682901782	0.1702469427742689
0.7514322022161295	0.7067051307290979	0.2896709436024730
0.0797317385655847	0.5723861522448693	0.0079518589654952
0.9395582100057130	0.6984859488201176	0.2526453264754265
0.2603702182922724	0.5510784393705125	0.0452132954223380
0.6188046991096979	0.6539329839911097	0.2416780040401564
0.3849357416073528	0.8555765328553687	0.7054754437510476
0.3707839265530714	0.9686715035098787	0.5601180851280300
0.3753791822956608	0.9360712366462520	0.6896351288487511
0.3904616472551768	0.8073866972777161	0.5914486451245762
0.3881834932922903	0.8393795149003587	0.4613666914754888
0.1634703571436710	0.9407051965222238	0.4537395904407039
0.2885610203077712	0.0843760651848753	0.3570167899272970
0.0400912630038178	0.0023236047571655	0.4418829626654507

0.4123561181068620	0.0216173864750888	0.3614031693165400
0.4701248248798521	0.9628863779198039	0.2657816169975734
0.7962274823368208	0.9775619129740579	0.3247642669695657
0.1706329385889067	0.9065568141966157	0.3422431527683380
0.6492568350896927	0.9845984629662112	0.2305539899606019
0.1039790611048265	0.0749150764786849	0.3939774217009843
0.4942239729078140	0.8629272941372476	0.3298447135507442
0.2513057863002586	0.7932782995025319	0.2103766756692161
0.5796930016707555	0.9275831478688443	0.4920279313894055
0.4393964359644806	0.8014379642748718	0.2475314118865382
0.7603356470541969	0.9488933790630942	0.4547773030723875
0.1187227138858834	0.8461189315786120	0.2582382444791049
0.1149899835940861	0.1444178011898320	0.2055073988005719
0.1292655276223872	0.0313351102681870	0.0601267395213883
0.1246578647271205	0.0639263209997270	0.1896507251520381
0.1093469664215470	0.1926117666939888	0.0914944608437210
0.1115378171138535	0.1606204744224277	0.9613960822616407
0.3364984080968189	0.0593511150624886	0.9538053585864578
0.2115483169669412	0.9156501132947480	0.8571089988865587
0.4599324088162125	0.9977434071287826	0.9419794369999940
0.0877011089913564	0.9783954392019949	0.8614678061061548
0.0298514079749848	0.0371153647953751	0.7657907252485767
0.7037427031527836	0.0224366824883617	0.8247517254864291
0.3292992608808827	0.0934758896988817	0.8422913727629956
0.8507168502670603	0.0154135691781779	0.7305446306644789
0.3961168068099989	0.9251442169709595	0.8940763997588488
0.0056548736232237	0.1370284015327276	0.8297532418101646
0.2485682890202826	0.2067052197944659	0.7103292951437252
0.9202682781626845	0.0723861881457711	0.9920482173117550
0.0604422052143365	0.1984862194081738	0.7473549197093404
0.7396298878449408	0.0510783047139163	0.9547867949900417
0.3811955206526927	0.1539330227916493	0.7583220818766752
0.1150641080927464	0.3555765056210644	0.2054754925966888
0.1292159847753546	0.4686714879469890	0.0601181614356274
0.1246206380579949	0.4360712155861226	0.1896352030701975
0.1095380983169353	0.3073866855369453	0.0914486669755094
0.1118162426099168	0.3393795246429039	0.9613667334490639
0.3365296300038202	0.4407052455980782	0.9537398076314311
0.2114388789765470	0.5843760431108088	0.8570167131723220
0.4599086112260718	0.5023237077585772	0.9418831756983245
0.0876438439521217	0.5216173622890937	0.8614030866417736
0.0298751233539295	0.4628863367658394	0.7657815757989657
0.7037724968150677	0.4775619119957901	0.8247642708950421
0.3293671675693412	0.4065568790116482	0.8422432600855053

0.8507431292916133	0.4845984697571039	0.7305539943575443
0.3960207363010486	0.5749151714603331	0.8939773976002078
0.0057761510773651	0.3629273014834946	0.8298446712754217
0.2486944506719438	0.2932783320468978	0.7103768701837854
0.9203069744296180	0.4275831180590256	0.9920278845951214
0.0606038447501704	0.3014379466960883	0.7475313965907852
0.7396643339325965	0.4488933354826957	0.9547772691400020
0.3812774594983093	0.3461189759539541	0.7582383651005179
0.3850098023122551	0.6444177370898490	0.7055075111167197
0.3707346037437893	0.5313351453179689	0.5601267363606049
0.3753421443492996	0.5639262692341176	0.6896507570803710
0.3906524440019997	0.6926117434810586	0.5914946384251123
0.3884618299303808	0.6606204745524741	0.4613962310066720
0.1635013116273620	0.5593511422882371	0.4538048381147002
0.2884523000812041	0.4156500700277798	0.3571093602561111
0.0400674252082726	0.4977434032819361	0.4419786235516774
0.4122991439316444	0.4783954490262431	0.3614683534598468
0.4701486867303520	0.5371153235393805	0.2657908250599004
0.7962575148227283	0.5224367881691656	0.3247518647343214
0.1707009383549934	0.5934760077823144	0.3422916195763993
0.6492833684704097	0.5154135747331526	0.2305448108456276
0.1038834392999109	0.4251441494537815	0.3940762344422368
0.4943454601560085	0.6370283732749368	0.3297531789556315
0.2514319819224617	0.7067050745833743	0.2103288639795658
0.5797319164460455	0.5723861027189749	0.4920483672133024
0.4395581534198119	0.6984860163748237	0.2473544948410048
0.7603702135751848	0.5510783294569975	0.4547869893525958
0.1188046912782024	0.6539330197820578	0.2583221385778269
0.8849356263066460	0.8555763873076018	0.7945244558927919
0.8707838106529343	0.9686714794353285	0.9398816898713092
0.8753790197395858	0.9360711407708867	0.8103646881753749
0.8904614518649969	0.8073866378666011	0.9085513557865579
0.8881834538738342	0.8393795791661319	0.0386331290477877
0.6634705056269032	0.9407051559539790	0.0462601398066238
0.7885610018790792	0.0843759490202896	0.1429834260003046
0.5400913953648291	0.0023235721689107	0.0581169288938358
0.9123561902324731	0.0216173183547063	0.1385969929386791
0.9701250278581753	0.9628863347220176	0.2342183557090965
0.2962276219658519	0.9775618760251321	0.1752354733710752
0.6706330377529028	0.9065568884303991	0.1577567579779238
0.1492571394444377	0.9845984672539686	0.2694459540168785
0.6039791604134829	0.0749150241294396	0.1060227614037285
0.9942239549565856	0.8629272217370353	0.1701552712754318
0.7513055967181699	0.7932783910567361	0.2896233785212860

0.0796929690366960	0.9275832307578105	0.0079719095242485
0.9393962489408703	0.8014379961251813	0.2524687382079924
0.2603355770291162	0.9488934635825256	0.0452223399123766
0.6187226569846808	0.8461190464354955	0.2417617335516453
0.6149900316696063	0.1444177584498762	0.2944927007677284
0.6292653804459082	0.0313350817770035	0.4398733654439763
0.6246576361477595	0.0639263044692367	0.3103493761776348
0.6093475623747280	0.1926117561283439	0.4085056126111405
0.6115383499217704	0.1606204687596575	0.5386040224161126
0.8364985942881976	0.0593511873528243	0.5461949565405881
0.7115482434530487	0.9156500819656163	0.6428907482539234
0.9599326227053860	0.9977434303893280	0.5580209256114783
0.5877011396004731	0.9783954357513182	0.6385320369151695
0.5298514920137961	0.0371153752571971	0.7342093834263592
0.2037426112509633	0.0224367222351959	0.6752484072840279
0.8292990503238676	0.0934759895026746	0.6577085976182681
0.3507168154065612	0.0154136201446800	0.7694555094297496
0.8961167548274833	0.9251442100135473	0.6059235426902569
0.5056545746295913	0.1370283494381644	0.6702468639578928
0.7485679603506712	0.2067051297130771	0.7896711477817527
0.4202681972093187	0.0723859762399398	0.5079518214990866
0.5604418324471481	0.1984860982094261	0.7526453572553027
0.2396298124629490	0.0510781856737219	0.5452132133893853
0.8811952656404832	0.1539330212762493	0.7416780628994386
0.6194312799723943	0.4297795731876846	0.5918437898931161
0.8806217188064293	0.5702311163455513	0.0918228396678323
0.3805686331817452	0.9297797227512744	0.4081559752458910
0.1193782688944856	0.0702311647856155	0.9081773086319589
0.1194313745093227	0.4297797332426464	0.9081560367868717
0.3806218538937760	0.5702311913049612	0.4081773373104561
0.8805688132686148	0.9297796944361130	0.0918439504696195
0.6193781911768433	0.0702311464406405	0.5918227900663653

H_2-Cu001 in POSCAR format

Note that the fragmentation we used comprised the Cu(001) surface in the first fragment and the H_2 molecule in the second fragment.

```
Cu H
   1.000000000000000
    7.657900   0.000000   0.000000
    0.000000   5.105300   0.000000
    0.000000   0.000000  25.000000
Cu   H
12    2
Direct
  0.000000  0.000000  0.000000
  0.166664  0.750005  0.927800
  0.000000  0.500010  0.000000
  0.166664  0.249995  0.927800
  0.333329  0.500010  0.000000
  0.500007  0.249995  0.927800
  0.333329  0.000000  0.000000
  0.500007  0.750005  0.927800
  0.666671  0.500010  0.000000
  0.833336  0.249995  0.927800
  0.666671  0.000000  0.000000
  0.833336  0.750005  0.927800
  0.050928  0.750005  0.064000
  0.949072  0.750005  0.064000
```

H_2-Pd001 in POSCAR format

Note that the fragmentation we used comprised the Pd(001) surface in the first fragment and the H_2 molecule in the second fragment.

```
Pd H
   1.000000000000000
    8.3900000000000006    0.0000000000000000    0.0000000000000000
    0.0000000000000000    5.5933000000000002    0.0000000000000000
    0.0000000000000000    0.0000000000000000   25.0000000000000000
  Pd   H
   12    2
Direct
 0.0000000000000000  0.0000000000000000  0.0000000000000000
 0.1666626936829587  0.7499150769670848  0.9209000000000032
 0.0000000000000000  0.5000089392666212  0.0000000000000000
 0.1666626936829587  0.2499955303666894  0.9209800000000001
 0.3333253873659103  0.5000089392666212  0.0000000000000000
 0.5000119189511310  0.2499955303666894  0.9209800000000001
 0.3333253873659103  0.0000000000000000  0.0000000000000000
 0.5000119189511310  0.7500044696333106  0.9209800000000001
 0.6666746126340897  0.5000089392666212  0.0000000000000000
 0.8333373063170413  0.2499955303666894  0.9209800000000001
 0.6666746126340897  0.0000000000000000  0.0000000000000000
 0.8333373063170413  0.7500044696333106  0.9209800000000001
 0.0464839094159686  0.7500044696333106  0.0640000000000001
 0.9535160905840314  0.7500044696333106  0.0640000000000001
```
